# Mobility and Income: Policy Insights to Support Public Electric Vehicle Charging Access

**DOI:** 10.1021/acs.est.6c02101

**Published:** 2026-06-29

**Authors:** Siobhan Powell, Marie-Louise Arlt

**Affiliations:** † Group for Sustainability and Technology, 27219 Swiss Federal Institute of Technology Zurich (ETH Zurich), Weinbergstrasse 56/58, Zurich 8092, Switzerland; ‡ Department of Law, Economics, and Business Administration, 26523 University of Bayreuth, Universitätsstrasse 30, 95447 Bayreuth, Germany; § Bavarian Center for Battery Technology, University of Bayreuth, Weiherstrasse 26, 95448 Bayreuth, Germany; ∥ ifo Institute, Poschingerstrasse 5, 81679 Munich, Germany

**Keywords:** electric vehicles, charging stations, policy, low income, equity, access, public charging

## Abstract

Widespread electric vehicle use is a key step to tackling transportation emissions, but adoption has yet to take off beyond high-income communities. A lack of access to public charging stations can be a major barrier to adoption. In this paper, we analyze the distribution and typology of public charging stations found in low-, mid-, and high-income communities across the US. Our results describe a robust neighborhood advantage, where high income in the surrounding area coincides with higher numbers of public stations in lower income block groups. We further show that station types in lower income areas are associated with shorter stops, suggesting they are designed to serve pass-through demand. Future income-based policies for public charging infrastructure should consider how new stations fit local residents’ mobility patterns and should use both local and neighborhood income to better target their support toward equal access for all future electric vehicle drivers.

## Graphical abstract

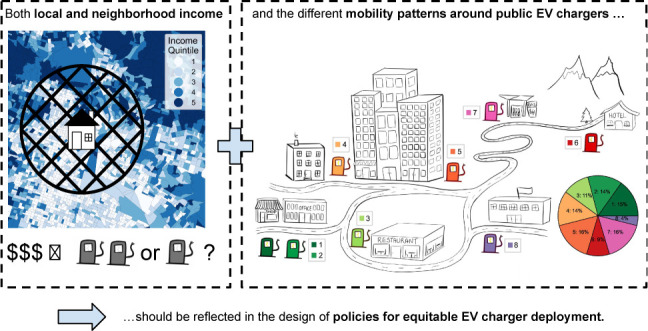

## 1 Introduction

Battery electric vehicles (EVs) are central to global plans to reduce greenhouse gas emissions from personal transportation.^1^ While multiple policies exist or have existed that directly subsidize vehicle purchase prices, including examples across Canada, the United States, Europe, and Asia,^2^ some research has found that policies that increase the number of available charging stations may be as or even more effective at supporting EV adoption.^3,4^ Convenient charging is critical to a driver’s decision to adopt and keep an EV,^5,6^ and the availability of public charging plays a particularly important role in adoption by creating positive network effects and norms.^7–10^

Early EV adoption has been concentrated in higher-income communities in many countries,^11,12^ but adoption must expand to all income groups to reach the climate and health benefits of widespread electrification.^13–15^ In the US, mid- and low-income drivers in particular face many barriers to EV adoption, including high upfront purchase prices, difficulty accessing rebate programs, a limited used vehicle market, and, critically, a lack of charging options.^13,16,17^ Home charging is not possible for many low-income and some mid-income drivers, who are more likely to be renters and residents of multifamily homes^17,18^ or face lower distribution grid capacities.^19,20^ Targeted policies can help address some of these barriers to home charging,^21^ but public charging options will be critical for many future EV drivers.

A growing number of policies acknowledge the need to extend the charging access to more drivers. Some target an even geographic coverage of public charging stations,^14^ both for slow charging within cities^22^ and for highway-side fast charging to enable long-distance road trips.^23,24^ Extensive research based on mobility patterns has optimized station placement to meet demand,^25,26^ but has not explored the connection with income.

Recent policy innovations have explicitly targeted funding for public charging stations toward low-income or disadvantaged communities (e.g., the Bipartisan Infrastructure Law in the US,^23^ see Supporting Information Note 1). This raises an important new research question, which we aim to address in this paper: are such income-based policies suitable to address gaps in charging networks and extend access to mid- and low-income drivers?

Previous research has provided mixed evidence of the correlation between charging access and income. In the US, a positive relationship has been found between income and access to public charging in U.S.-wide analyses of household-level data^27^ and census tract-level data,^28^ but the relationship can be positive or negative for different states, counties,^27^ and cities. Early research in California found a similar distribution of public charging stations between disadvantaged and non-disadvantaged communities,^29^ but more recent data have revealed growing inequality and lower access to public charging stations for low-income communities.^30,31^ Higher access to public charging has been associated with higher income in New York City and Chicago,^32,33^ but lower income in Seattle.^34^ Mixed evidence has also been found in countries beyond the US.^35,36^

These contradicting findings could be explained in part by local differences in settlement and mobility patterns. A charging station in a low-income neighborhood may serve demand from outside the community, and local residents may also charge in other areas. Recent literature has highlighted the relevance of some of these factors. The presence of highways is strongly associated with increased public fast charging.^31,32,37^ Co-location with different types of points of interest can also impact charging station placement and utilization patterns.^38,39^ In particular, commercial land use and the presence of recreational points of interest have both been associated with more public charging.^33,34,40,41^ Neighborhoods and spatial spillovers can also play a role. Evidence from Seattle, for instance, suggests that access to charging is correlated for neighboring census tracts.^34^ Individual mobility trajectories also affect individuals’ access to different public and workplace charging options, with substantial spatial disparities across neighborhoods.^42^ It is unclear, however, how such mobility-related factors interact with income-based policies for access to charging.

In this paper, we combine US-wide data on mobility patterns, public charging stations, and socioeconomics to fill this gap in our understanding and analyze the relationships among station placement, station use, and income. By considering travel patterns and effects related to the stations’ neighboring communities, we help explain the mixed results of previous research and provide guidance for the design of income-based policies for EV charging station deployment. First, using a regression analysis on data from across the 50 US states and the District of Columbia, we show that higher station counts are positively correlated with lower income in the local community and higher income in the surrounding area. Second, we use clustering to derive a data-driven typology of US public charging station locations based on a mix of socioeconomic and mobility pattern data. We find that the types of stations located in high-, mid-, and low-income communities differ, with stations in low-income areas more likely to serve the demand of drivers making short stops. Third, we compare results at the state level and find that states with higher shares of EVs have stronger local and neighborhood advantages and higher shares of station types that serve local charging needs. Our results offer new insights that are relevant for policymakers around the world. We recommend that future policies consider both local and regional income levels, not local income alone, and that future policies consider local mobility patterns to ensure that charging stations built in low- and mid-income communities are useful, accessible, and affordable for local drivers.

## 2 Materials and Methods

We use multiple data-driven methods to investigate these research gaps: we use exploratory data analysis and present summary statistics on the relationship between income and number of stations; we use regression analysis to confirm these relationships, both in the U.S.-wide data and for individual states or types of stations; and we use clustering to identify different types of stations based on their placement and local mobility patterns.

For our main analyses, we leverage two data sets: a block group-based data set and a charging station-based data set, both of which cover the 50 US states and the District of Columbia. A block group (BG) is a small geographic unit defined by the US Census Bureau.^43^

The following section introduces the data and methods used for our analysis. Table 1 summarizes the methods and data used to generate each result.

**Table 1:** Summary of the Methods and Data Use for Each Piece of Analysis

Analysis	Method	Data set	Result
Number of stations by income quintile (US)	Summary statistics by BG and county income quintiles (US)	BG-level	Figure 1d
Number of stations by income quintile (state)	Summary statistics by BG income quintiles (state)	BG-level	Figure 1e
Relationship between income and number of stations	Regression analysis with state fixed effects	BG-level	Table[Table tbl2]
Relationship between within-county relative household income quintile and public charging stations	Summary statistics by within-county relative household income quintile	BG-level	Figure 2c
Distribution of clusters	Summary statistics by cluster	Station-level	Figure 3b
Distribution of station characteristics by cluster	Summary statistics	Station-level	Figure 3c
Distribution of clusters by income quintiles	Summary statistics by income quintile	Station-level	Figures 4a-c
Relationship between income and number of stations by cluster	Regression analysis with state fixed effects	BG-level	Figure 4d
Distribution of clusters by US state	Summary statistics by state	BG-level	Figure 5a
Relationship between cluster share and socioeconomic characteristics	Univariate regression	State-level	Figure 5b
State-specific relationship income and number of stations	Regression analysis	State-specific BG-level	Figures 6a-b
Relationship between coefficients and EV stock	Univariate regression	State-level	Figure 6c

### 2.1 Data Sources

We compile the block group-based and charging station-based data sets by pulling together data from multiple different sources. First, we use charging station data provided by the US Department of Energy through the Alternative Fuels Data Center (AFDC), downloaded on April 03, 2023.^44^ In this data, and in the language used in this paper, a single “charging station” may host multiple charging plugs. The data set includes 59,939 entries for electric vehicle charging stations in the US. Each entry includes the station’s geolocation, number of charging plugs, owner or network operator, pricing, and technical characteristics. We assume all stations in the data are public or semipublic based on their inclusion in the data set. We use administrative geodata shapefiles provided by the US Census Bureau^43^ to assign a FIPS code to each station based on its geographic coordinates. We keep all stations which can be successfully assigned to the 50 states of the US or the District of Columbia. The final data set contains 59,807 charging stations and dates from before new income-based policies, such as the 2022 Bipartisan Infrastructure Law, had a wide effect on deployment. Specifically, there was no need to specifically filter out stations affected by the BIL from the April 2023 station data because, even by early 2024, almost no BIL-funded stations had yet been deployed, largely due to delays in state-level implementation plans.^45^ Finally, we classify a station as free-of-charge if the non-structured pricing information includes the word “free”. Unfortunately, we are not able to analyze station types over time for a given state, as the data quality is poor with regard to the stations’ opening dates.

We further use socioeconomic data from the US Census and the American Community Survey on the block group level as of 2020, as provided by SafeGraph.^46^ The data include BG-level mean household income, total population, race, commuting mode and duration, and household size. Supporting Information Table S9 details the full set of variables considered. We combine these data with information from the US Council on Environmental Quality’s Climate and Economic Justice Screening Tool (CEJST) for the classification of census tracts as disadvantaged.^47^

To account for travel patterns at and around charging stations, we use mobility data provided by the company SafeGraph.^46^ The mobility data consists of aggregate patterns on the point of interest (POI) level for 12,148,516 POIs across the country and we use average mobility patterns for the month of October 2022. POIs include all types of commercial and public sites, including sites like stores, restaurants, schools, offices, hospitals, parks, and train stations. In particular, for each POI, the data includes a count of visitors by the time of day (binned in 3 h windows) and by the day of the week, the average dwell time (absolute and binned), and a measure of the POI visitors’ average distance from home. We further map the POI categories to a set of ten higher level categories (see Supplementary Table S10). These categories are Commercial, for all types of stores and sites with similar visitation patterns like post offices and car rental companies; Manufacturing, for all sites factories, industrial sites, and sites with similar visitation patterns like agriculture or forestry; Recreation, for sites like parks and museums; Education/Child Care, for schools, day cares, universities, and other sites with similar purposes; Transit, for sites like transit stations or ferry terminals; Gas Station, for gasoline stations; Medical, for hospitals, clinics, and doctor’s offices, as well as residential care facilities and other community health sites; Office, for all non-customer-facing workplaces where workers are likely to have standard office hours, like accounting and architecture firms; Hotels, for traveler and temporary accommodations; and Restaurants and Bars, for public eating and drinking places.

We finally use state-level data on the number of private vehicles (total and battery electric) in 2023, as provided by the AFDC.^48^ We further use shapefiles of primary and secondary roads, including highways, provided by the US Census Bureau.^49^

### 2.2 Block Group-Based Analysis

We use several approaches to analyze the relationship between the number of charging stations and local income at the BG level. Here, we define local income as the median household income within a BG.

#### 2.2.1 Data

Our BG-level analyses are based on a BG-level data set covering all BGs in the 50 US states and the District of Columbia. The data set contains BG-level Census data described in section 2.1. We further use the median household income data to compute the population-weighted county-level household income and the Gini coefficient by state. We assign the BGs and counties to income quantiles in 20% steps (quintiles), pooling all BGs or counties in the data set to calculate US-level quintiles, then pooling BGs and counties by state to calculate state-level quintiles. As the District of Columbia has just one county, we set its state-level county income quintile to 3. To avoid bias because of missing values, we impute missing BG values based on the population-weighted values of the other BGs in the same census tract. We use these imputed values throughout the analysis, including in the calculations of BG- and county-level income quintiles.

In addition, we compute the neighborhood income for each BG as the population-weighted median household income for all BGs at all touched by a 10 km or 50 km buffer around the BG, excluding the local BG itself. The 10 and 50 km distances were chosen because they capture over 51% and 93% of vehicle trips.^50^ We calculate the minimum distance of each BG to the nearest primary and secondary road based on the BG edges; the distance is 0 km if a primary or secondary road intersects with the BG. We further include the number of charging stations and POIs within each BG, whether a BG is disadvantaged according to the definition provided by the US Environmental Protection Agency, and the share of neighboring BGs that are disadvantaged.

#### 2.2.2 Regression Analysis

First, we use regression analysis to quantify the overall relationship between income and charging stations. For our main specification, we regress the number of stations in each BG, *N*_*BG*_, against the local and neighborhood income, *I*_*BG*_ and *I*_*BG*_^10*km*^, in units of 10,000 USD, using ordinary least-squares (OLS) regression with state-fixed effects:

1 $$N_{BG}=\alpha +\beta^{Local}I_{BG}+\beta^{Neighborhood}I_{BG}^{10km}+\gamma \mathrm{StateFE}+\epsilon_{BG}$$

We label the local and neighborhood coefficients β_*Local*_ and β_*Neighborhood*_ which describe the relationship between the number of stations and local and neighborhood income, respectively. We do not adjust for the population in each BG in this regression because BGs are delineated by the U.S. Census Bureau with the aim that they all contain a similar number of people. We include a robustness check using population weights in Table S3.

Note that our regressions are only used to test for the statistical relationship between stations and income and do not allow for a causal interpretation. In particular, it may suffer from endogeneity, i.e., local income may increase as a result of station placement. We do, however, believe that these effects are limited as our income data are from 2020, while 50.1% of charging stations were installed in 2021 or later (including April 2023, the last month of our data). For robustness, we also include a regression in Supporting Information Table S5 where we use income data from the 2014 American Community Survey.^51^ 92.1% of charging stations in the data were installed in 2015 or later.

We further consider alternative specifications, including a regression with local income only as well as the neighborhood income within a 50km radius, *I*_*BG*_^50*km*^. For estimation, we use the statsmodels Python package.^52^

#### 2.2.3 Charging Stations by Within-Region Income

Second, we define the “within-county relative income percentile” to systematically compare relatively higher- and lower-income communities within a smaller region. This metric is calculated by ranking the BGs within each county by median household annual income. We compute the average number of stations by “within-county relative income percentile” to derive the distribution of stations by relative income within a county.

#### 2.2.4 State-Level Analysis

Finally, we repeat our regression analyses separately for each state to analyze station cluster-specific coefficients:

2 $$N_{BG}=\alpha^{c}+\beta^{Local,\mathrm{state}}I_{BG}+\beta^{Neighborhood,\mathrm{state}}I_{BG}^{10km}+\gamma^{state}\epsilon_{BG}$$

We further analyze the correlation of the neighborhood coefficient with different state-level characteristics, including the level of EV adoption, income inequality as measured by Gini and Moran’s I, and the presence of disadvantaged communities as used by the 2022 Bipartisan Infrastructure Law.

### 2.3 Station Clustering and Station-Based Analysis

We use clustering to derive a typology of US public charging station locations based on a mix of socioeconomic and mobility pattern data. We leverage the typology to describe differences in station type distribution in communities across different income levels.

#### 2.3.1 Data

In preparation for the clustering, we compile a station-level data set based on the AFDC charging station data. We include socioeconomic BG-level data based on each station’s location. We calculate the distance from each station to the nearest primary or secondary road. Leveraging the SafeGraph mobility data, we then calculate the number of POIs of each type (Commercial, Manufacturing, Recreation, Education/Child Care, Transit, Gas Station, Medical, Office, Hotels or Restaurants, and Bars) located within 500 m of each station, excluding charging station POIs. We count nearby stations based on the charging station data.

To approximate the mobility patterns at a given charging station location, we calculate the mean of the mobility properties over all POIs in the data set within a 500 m radius of the station. We use mobility properties from the SafeGraph mobility data set for each POI including: a normalized count of visitors by time of day and by day of the week, a distribution of visitor dwell times, and a measure of visitor’s distance from home (listed in full in Supplementary Figure S8). For stations with fewer than 10 POIs in the data set within that radius, we use the closest 10 POIs within a 5 km radius. 320 or 0.535% of the stations in the data set had no mobility pattern data based on these calculations, either because there were no POIs within 5 km or because pattern data was not available at nearby POIs. We fill those missing values with the data set average to ensure they are not deciding values in those stations’ clustering. We normalize the visitor counts by time of day or day of the week over the total visitors to each POI.

#### 2.3.2 Clustering

For the clustering analysis, we use 52 metrics in total to describe each station’s location: the number of other public stations within a 500 m radius; the BG-level average number of rooms per housing unit, number of housing units per person, share of owner-occupied housing and of detached houses, population density, share of the population identifying as white, share of the population that commutes by car, by public transit, or by active commute, share of the population with commutes between 0 and 24 min, between 25 and 44 min, or 45 min or longer, and share of the population with some level of education beyond high school; the total number of POIs in the block group, the share of each POI category among those POIs, the nearby POIs’ visitors’ average distance from home and dwell times at nearby POIs, and the popularity of nearby POIs by weekday and time of day.

We apply a power transformer with the Yeo-Johnson method and a zero-mean, unit-variance normalization to prepare the data for clustering.^53^ We then apply K-Means clustering to the station locations.^54^ We consult the elbow curve of within-cluster inertia to select the number of clusters as K = 8. We draw a trade-off between choosing a relatively stronger elbow and ensuring a large enough number of clusters to provide meaningful findings and interpretations for our analysis. A comparison with other selections of K and other types of clustering is presented in Supporting Information Note 8. We implement these methods using the sci-kit learn and pandas packages in Python.^55,56^

#### 2.3.3 Regression Analysis

We specify the regression from Section 2.2 to analyze station cluster-specific neighborhood coefficients:

3 $$N_{BG}^{c}=\alpha^{c}+\beta^{Local,c}I_{BG}+\beta^{Neighborhood,c}I_{BG}^{10km}+\gamma^{c}\mathrm{StateFE}+\epsilon_{BG}^{c}$$

where the dependent variable *N*_*BG*_^*c*^ corresponds to the number of stations of cluster *c* in *BG*.

## 3 Results

### 3.1 Relationship between Income and Charging Station Deployment

We first use the BG-level data set described in section 2.2 to provide descriptive evidence of the relationship among income, EV adoption, and the density of public EV charging stations. We find that the relationship is complex, with wide variations across the US at the county level. Figure 1 shows that some of the counties with the lowest average household income (Q1 in Figure 1a) are also the counties with the highest rate of public charging stations per person (Q5 in Figure 1b). Similarly, some states that have many counties with a very high density of public charging stations (Q5 in Figure 1b) are also states with relatively low EV adoption, like Wyoming or Michigan (Figure 1c).

**Figure 1:**
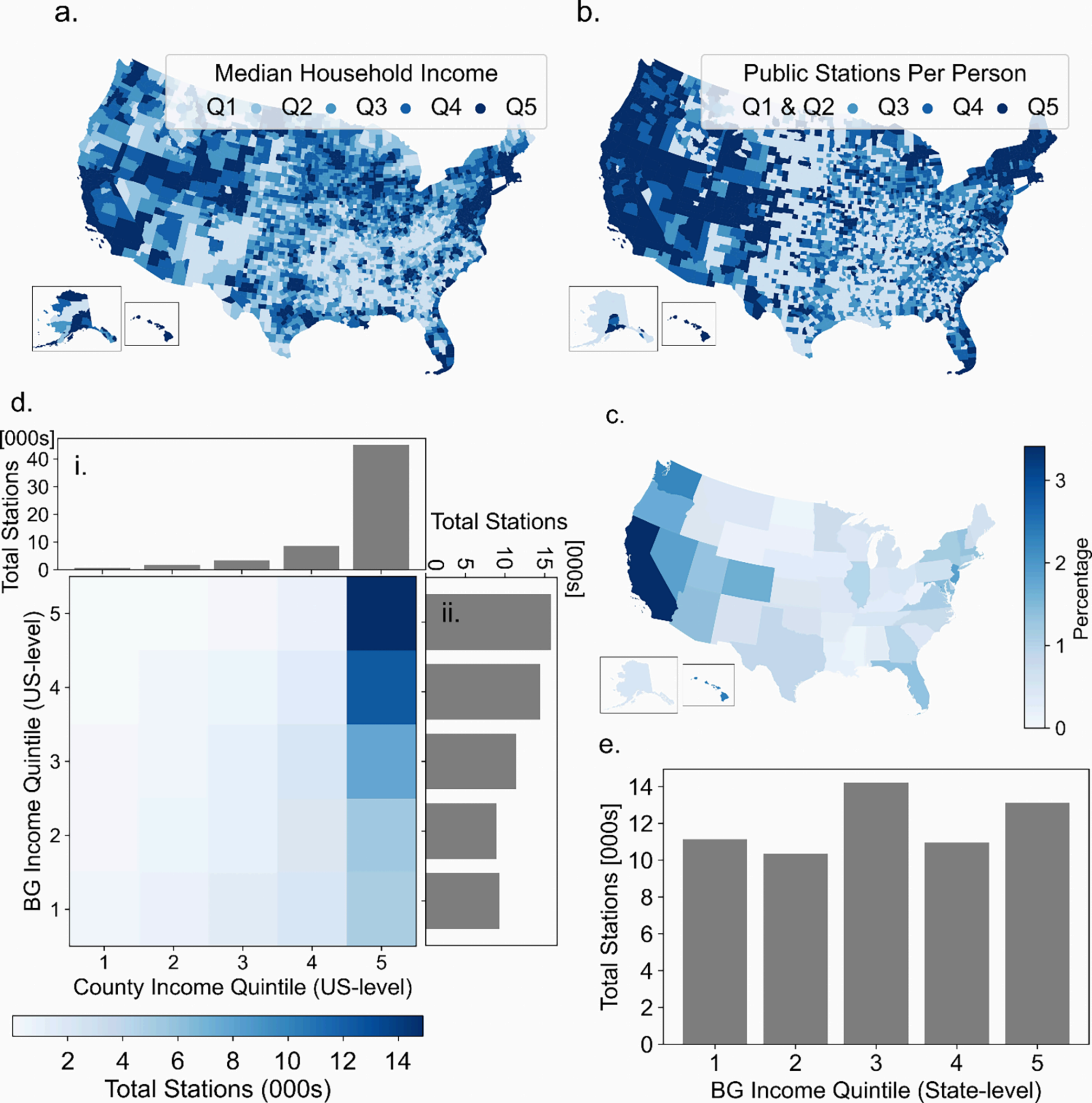
Aggregate relationship between income, EV adoption, and the locations of EV charging stations. Quintiles (20% steps) computed based on the distribution of all US counties are illustrated for (a) median household income and (b) the count of public charging stations per person in April 2023 (see Methods). Quintiles 1 and 2 are merged in (b) because more than 20% of the counties had zero stations per person. EV adoption is shown at the state level for the year 2023 (c). The count of public stations is broken down by block group (BG) and county income quintile in (d). We see that access to public charging stations relates more clearly to county-level income (d.i) than BG level income (d.ii). When block-group income quantiles are calculated at the state level (e), we see that stations are distributed nearly evenly across the five quintiles.

We analyze the number of stations by the income level in each local area to better understand this relationship. Figure 1d.i shows that the average number of public charging stations by county increases steeply with a higher county-level income. However, the picture changes when we zoom in to the block group (BG) level, a smaller geographic unit with an average population of 1374 residents (in BGs with populations greater than zero) and an average radius of 3.5 km. With BG income levels binned at the national level, shown in Figure 1d.ii, the skew of stations toward higher income levels is still there but is much less pronounced. With income levels binned within each state as shown in Figure 1e, we find nearly as many stations in low-, mid-, and high-income block groups. Supporting Information Figures S1–S4 confirm these patterns for different binning methods. These findings suggest that local income relative to income in the surrounding area has an important effect on station placement. If only local income or only county-level income was important for the count of stations, we would expect to see the same pattern within each row and column of Figure 1d. Instead, we see the count of stations increase with BG income within Q5 income counties but decrease with BG income within Q1–4 income counties.

The relevance of the surrounding neighborhood is further supported by regression analysis (Methods as described in Section 2.2). Table 2 summarizes the results. When we regress the number of public charging stations in a BG against only the median household income in the local BG, we see a significantly positive effect of local income (model a in Table 2). This confirms the broad findings in the literature. However, when we include the average income in other BGs touched by a buffer of 10 or 50 km around the BG (models b and c in Table 2), we instead see a significantly negative effect of local income, and the contribution of the surrounding neighborhood’s income is significantly positive and stronger. We call this positive effect of surrounding income the “neighborhood advantage”.

**Table 2:** Regression Analysis at the BG Level of the Relationship between the Number of Public Charging Stations, Local Income in the BG, and the Average Income Across Neighboring BGs Touched by a 10 km and 50 km Buffer around the Local BG (Excluding the Local BG Itself)^a^

	Dependent variable: Number of stations		
Independent variables	(a)	(b)	(c)
BG median household income [10k USD]	0.003*** (0.001)	–0.012*** (0.001)	–0.003*** (0.001)
Income 10 km around BG [10k USD]		0.056*** (0.002)	
Income 50 km around BG [10k USD]			0.054*** (0.003)
Observations	239780	239766	239776
*R* ^2^	0.010	0.013	0.012
Adjusted *R* ^2^	0.010	0.013	0.012
Residual Std. Error	1.643	1.640	1.641
F Statistic	48.536***	62.087***	55.224***

a Model (a) only considers the local effect. Model (b) represents our main specification (eq 1). Model (c) instead uses the neighborhood income within a 50 km radius. For Models (b) and (c), we see a significant positive effect of the average neighborhood income that comes with a significant negative effect of local income. Income refers here to median annual household income. All three analyses are with state fixed effects. The correlation between local and 10 km neighboring income is 0.59 (VIF for model without fixed effects: 6.99) and between local income and 50 km neighboring income 0.46 (VIF for model without fixed effects: 5.74). These values show moderate spatial correlation; however, the robustness of our estimates as well as the drivers and mechanisms presented in the subsequent analyses support the relevance of our results. Note: *p < 0.05; **p < 0.01; ***p < 0.001.

We use the term “neighborhood advantage” to distinguish from the existing term “neighborhood effect” which instead relates to individual behaviors.

We note that the strongly significant F statistics show that income contributes to explaining the number of stations in a BG, and the strongly significant coefficients show that income is correlated to the number of stations. These models are used only to test the statistical relationship, not to predict the number of stations; we focus only on the relationship between the charging station count and income to mimic purely income-based policies, with the model’s low *R* ^2^ as a result.

We confirm the presence of a positive neighborhood advantage with multiple robustness checks including Poisson or negative binomial specifications; repeating the analysis at the census tract level; with a regression for the presence of at least one station, or the exclusion of BGs without any stations; and using the population density, distance to the nearest highway, or the presence of a highway passing through the BG as additional covariates (see Supporting Information Tables S1–S4). In all cases, we found significantly positive effects of the neighborhood income. In all cases except one, this comes with significantly negative effects of the local income.

This finding suggests that local income alone is not a sufficient policy condition as higher income EV drivers can travel to stations in nearby neighborhoods. Low-income BGs within larger low-income areas may see the lowest number of stations because they do not benefit from this effect.

### 3.2 Neighborhood Structures and the Neighborhood Advantage

To investigate the source of the neighborhood advantage in more detail, we first provide descriptive evidence of the geographic distribution of different neighborhoods. High-, mid-, and low-income households are within easy driving distance of each other in many US cities. We observe high spatial heterogeneity in income at the block group level, a “salt and pepper” pattern. For the example of Los Angeles County, California, Figures 2a and 2b show several clusters of charging stations located in low- and mid-income block groups.

**Figure 2:**
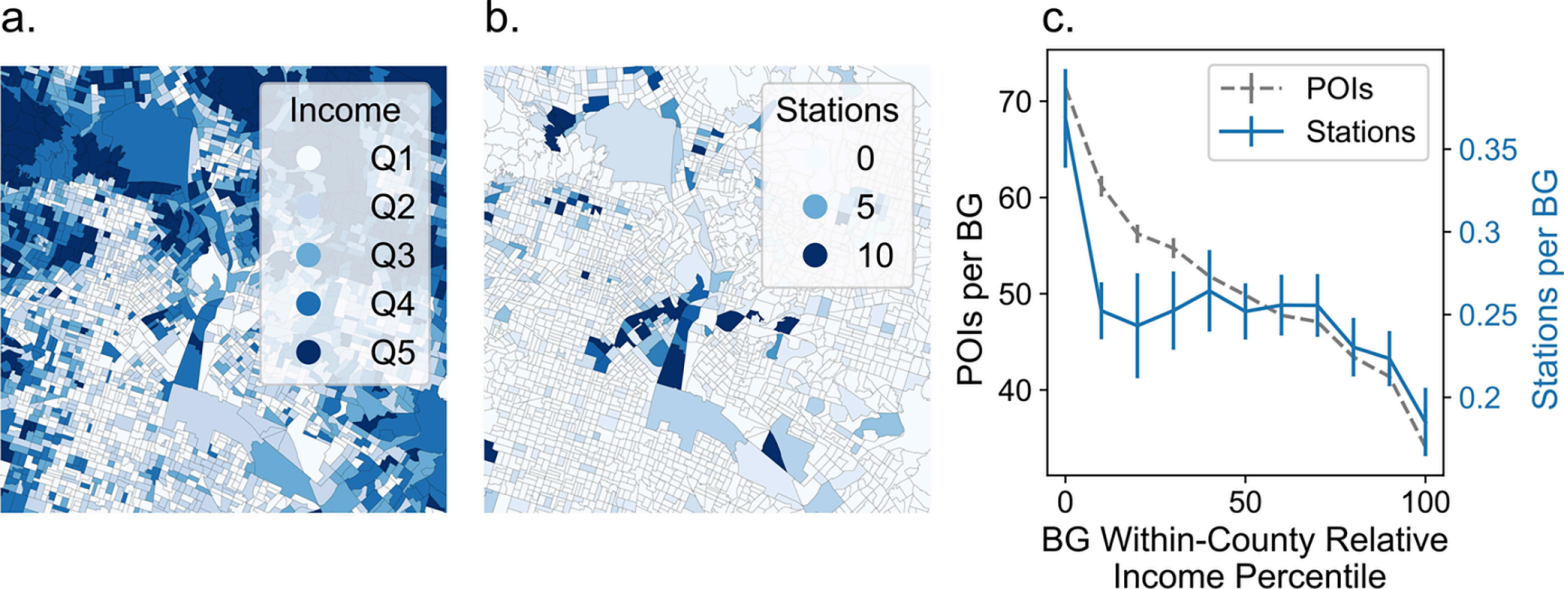
We illustrate the alignment or misalignment of (a) within-county relative household income quintile and (b) public charging stations for a section of Los Angeles County, California. Across all counties in the US-wide data set (c), we find a strong relationship between within-county household income level and the number of points of interest (POIs) and stations in a BG. In (c), error bars show the 95% confidence interval. In (a) and (b) we include a section of LA county centered on downtown Los Angeles, extending from Mount Hollywood and the Santa Monica Mountains in the upper left to the industrial areas around Commerce in the lower right.

We further compute the within-county relative income percentile as defined in section 2.2 to analyze the distribution of stations between higher- and lower-income communities within a smaller region. Figure 2c shows the average number of stations by within-county relative income percentile in BGs across the whole US. We find that the BGs with the lowest within-county relative income have both the highest number of public charging stations and the highest number of overall points of interest (POIs), which include sites such as restaurants, stores, offices, parks, and schools. This pattern is consistent across different types of POI (see Supporting Information Figure S5). As many public charging stations are co-located with existing POIs, which attract drivers from nearby areas, this may partly explain the high number of charging stations observed in low-income BGs in Figure 1e.

### 3.3 Typology of Charging Stations

Though there are many public stations in some low-income areas, those stations may differ from stations in high-income areas in ways that limit their accessibility to low-income residents.

We apply the clustering described in section 2.3 and identify eight distinct clusters. Figure 3a illustrates the cluster interpretations. The labels are derived from the characteristics of stations in each cluster shown in Supporting Information Figure S8. The descriptive labels for the eight station clusters are discussed in more detail in Supporting Information Table S5. We focus on mobility patterns that suggest how different station types are used. We find two clusters near POIs that are most visited in the middle of the day (clusters 1 and 2) and three clusters near POIs most popular in the evening or overnight (clusters 3, 5, and 6). We also find two city clusters in areas with high population density and a broad mix of POIs, but the City Neighborhoods stations (cluster 4) have visitors with much lower average distance from home than the City Destination stations (cluster 5; Supporting Information Figure S9). Most of the clusters are similar in size at the US level, except for the Hotels and University/College Campus station clusters, which are smaller but have highly distinct mobility patterns (Figure 3B).

**Figure 3:**
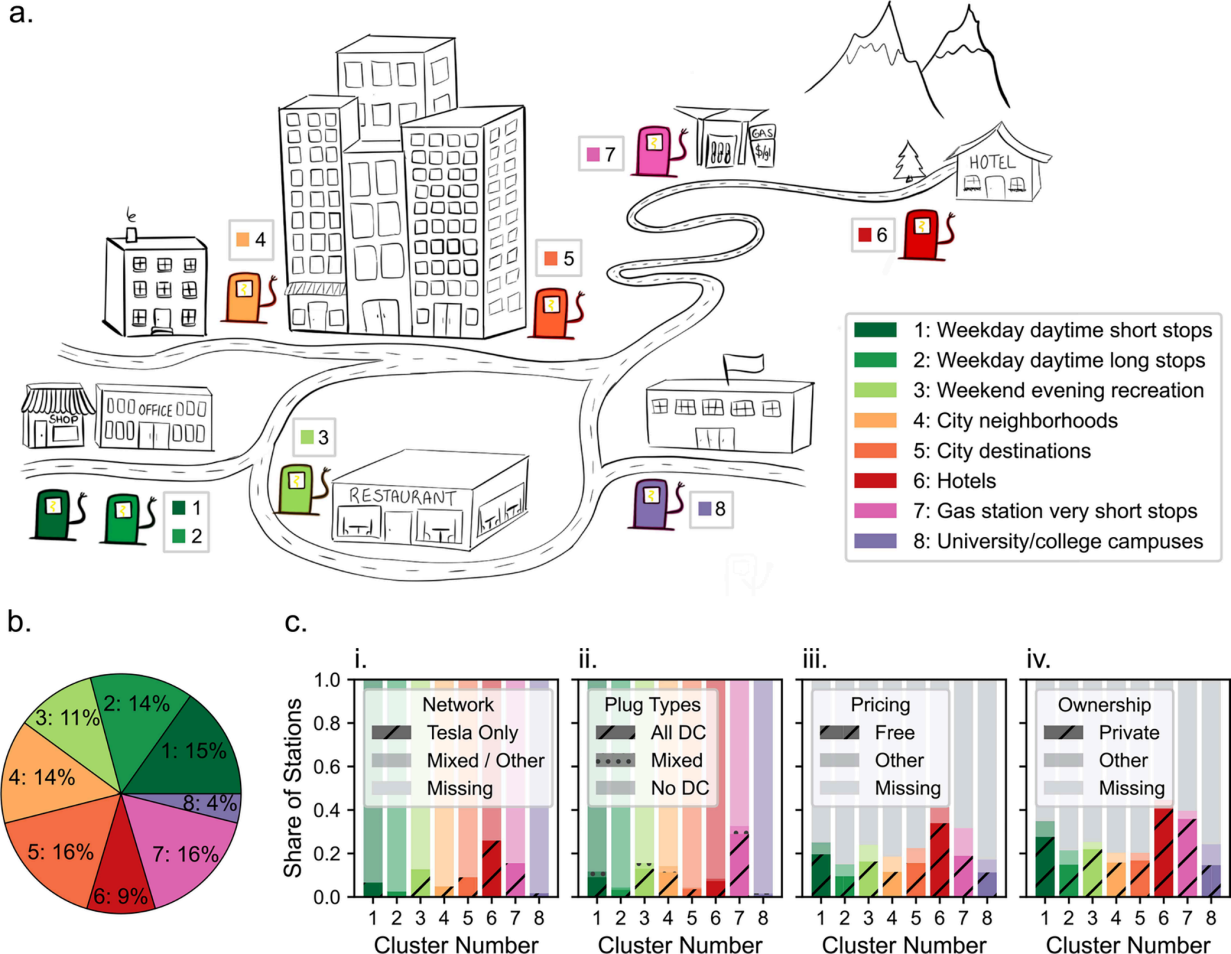
Eight clusters are identified based on mobility patterns at nearby points of interest and socioeconomic data at each station location. (a) shows an illustration of the cluster interpretations, (b) the distribution of stations across the eight clusters, and (c) statistics by cluster on stations’ accessibility (i), plug type (ii), pricing (iii), and ownership (iv). The legends in (c) refer only to the shading and hatches in the bars. The colors throughout this figure and throughout the remainder of the paper are each consistently associated with one of the eight station clusters. Charging in the Hotels and Gas Stations Very Short Stops clusters may have the most limited access for low-income residents. Image source: the illustration in (a) was drawn by the authors.

We further provide descriptive evidence with regard to each cluster’s accessibility, ownership, technical specifications, and pricing, as illustrated in Figure 3c. Stations in the Hotels, Weekend Evening Recreation, and Gas Station Very Short Stops clusters are more likely than others to have only Tesla-branded charging stations, which in 2023 were only accessible to drivers of higher-priced Tesla vehicles.^14^ Stations in the Gas Station Very Short Stop cluster were also more likely than others to have only DC fast-charging plugs, which are often more expensive than slower charging options.^57,58^ Among stations with data on pricing (24.5% of stations) and ownership (28.8% of stations), gas station very short stops stations were the least likely to allow charging for free and, with hotels stations, the most likely to be privately owned.

We use our typology to investigate the distribution of stations by BG-level income. Figure 4a shows our main result (for more details, see Supporting Information Figures S11 and S13). We find that the Hotels and Gas Station Very Short Stops clusters make up a much larger share of the stations in the lowest-income counties than in the highest-income counties. We saw previously that these clusters are also more likely to have exclusive or high-cost charging technologies (see Figure 3c). Conversely, we see that the highest-income counties host more charging stations of clusters more likely to serve local demand, including those with longer stops, those located in city neighborhoods, and those with lower cost, more accessible charging technologies. Supporting Information Figures S10 and S12 show the same patterns even more strongly with quintiles calculated at the US level. Together, these results may suggest that stations built today in lower-income communities target higher-income EV drivers passing through and are less accessible or affordable for local residents.

**Figure 4:**
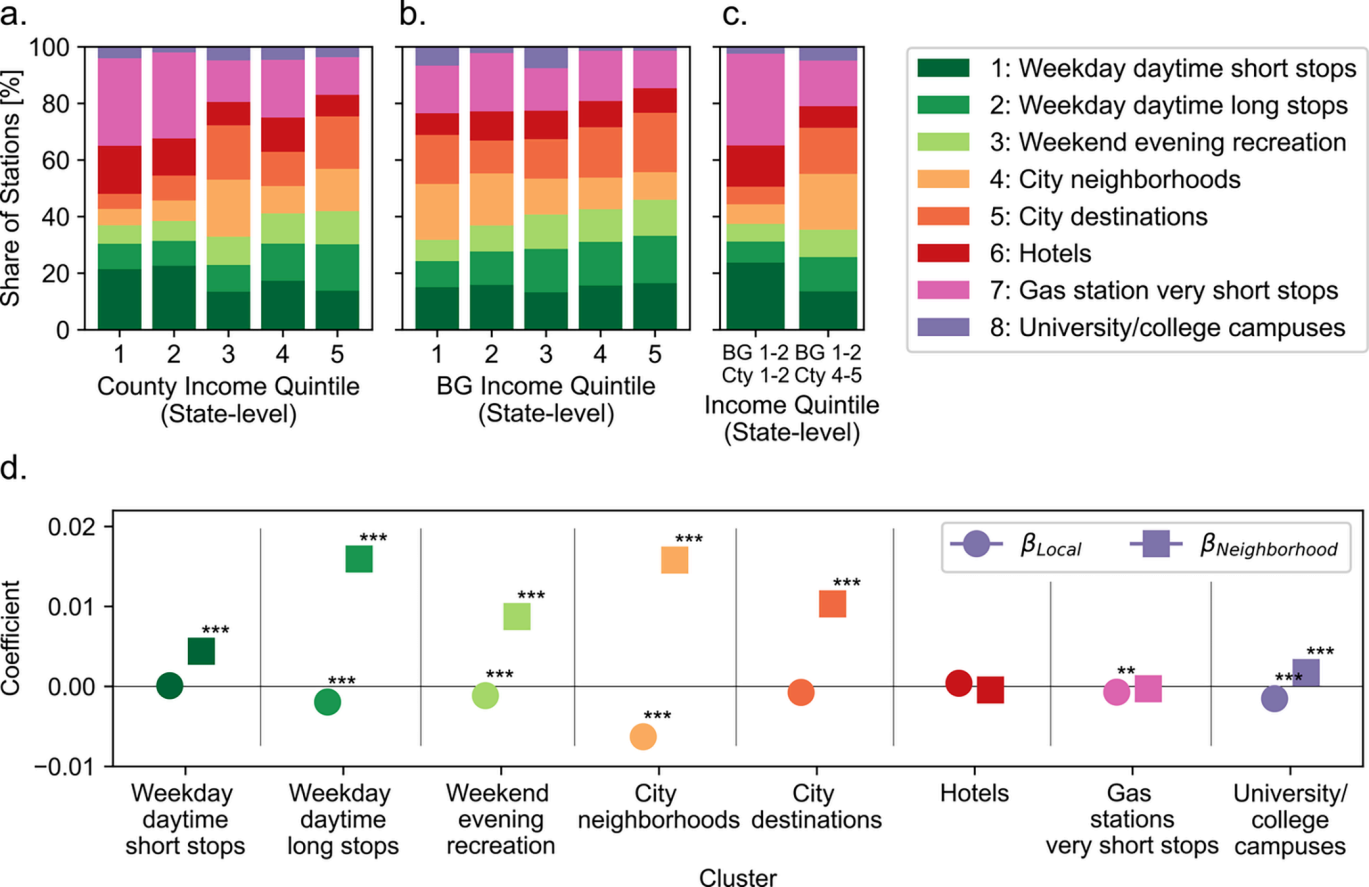
Share of stations in each cluster broken down by (a) county- and (b) BG-level income quintile, with quintiles calculated within each state. (c) The cross case for stations in lower income BGs (BG quintiles 1 and 2) and either lower income counties (Cty quintiles 1 and 2) or higher income counties (Cty quintiles 4 and 5). (d) The local and neighborhood regression coefficients for stations in each cluster; the vertical lines separating the clusters are used to indicate that the regression was run separately for each cluster. Stars indicate the p-values: *** for *p* < 0.001, ** for *p* < 0.01, and *for *p* < 0.05. The absence of stars indicates *p* ≥ 0.05. The sign and significance of the coefficients in (d) can indicate the presence of a positive or negative neighborhood advantage for stations in that cluster. The Hotels and Gas Station Very Short Stops clusters are overrepresented in low income counties and are the only two clusters without a significantly positive neighborhood advantage.

We apply the regression analysis described in section 2.3, now using the number of stations in each cluster as the dependent variable. In section 3.1, our regression of the count of stations in each U.S. BG showed a significantly negative effect of local income and a stronger, significantly positive effect of higher income in the surrounding 10 or 50 km area. We called this positive effect of the surrounding area income the neighborhood advantage.

In the results of the cluster-specific regressions shown in Figure 4d, the sign and significance of the local and neighborhood coefficients reveal the presence of either a positive or negative neighborhood advantage for stations in each given cluster. The magnitude of the significant coefficients may relate to the strength of the relationship between income and station presence in that cluster, but could also be influenced by other factors, such as spatial correlation, so should not be the focus of any interpretation. We identify a significantly positive neighborhood advantage for stations in the Weekday Daytime Long Stops, Weekend Evening Recreation, City Neighborhoods, City Destinations, and University/College Campuses clusters, which suggests that BGs benefit from demand in the surrounding areas for stations in these cluster types. This shows that stations from these clusters and the destinations with which they are co-located are situated nearby but slightly outside of higher income BGs where more EV drivers live. This empirical observation suggests a preference of high-income residents for separation between their homes and the non-residential POIs they frequent. We can hypothesize several possible mechanisms behind this finding, including a preference of high-income residents to avoid attracting more traffic and visitors to the neighborhood, limitations in zoning restrictions, or even the charging station operators’ decision to build on less expensive land nearby but not directly inside high-income BGs; these possibilities would need to be addressed with a causal analysis. The Gas Station Very Short Stops and Hotels clusters, on the other hand, see no significant effect from neighborhood income, which is consistent with the interpretation that those stations serve demand from further away.

### 3.4 State-Level Analysis and Connection to EV Adoption and Policies

We extend our analysis to the state level. Figure 5a shows that the distribution over clusters varies substantially from state to state (for more details, see Supporting Information Figure S14). Descriptively, Figure 5b illustrates that these differences are associated with state-level EV adoption. In states with very high EV adoption (which includes many coastal states), there are relatively more city stations and relatively fewer stations meant for short-term stops. This aligns with evidence of lower levels of adoption and charging station access in rural areas.^59^ More mature markets have more drivers with a wider range of preferences, like for workplace or destination charging,^60^ and driver profiles beyond those of early adopters, likely requiring more heterogeneous charging station types. In states with very low EV adoption (which includes many interior states), there are relatively more short stop and hotel stations. For example, in Wyoming, 38% and 32% of stations fall in the Hotels and Gas Stations Very Short Stops clusters, compared to only 7% and 9%, respectively, of stations in California. This may reflect stations meant to serve demand from early adopters or out-of-state drivers passing through.

**Figure 5:**
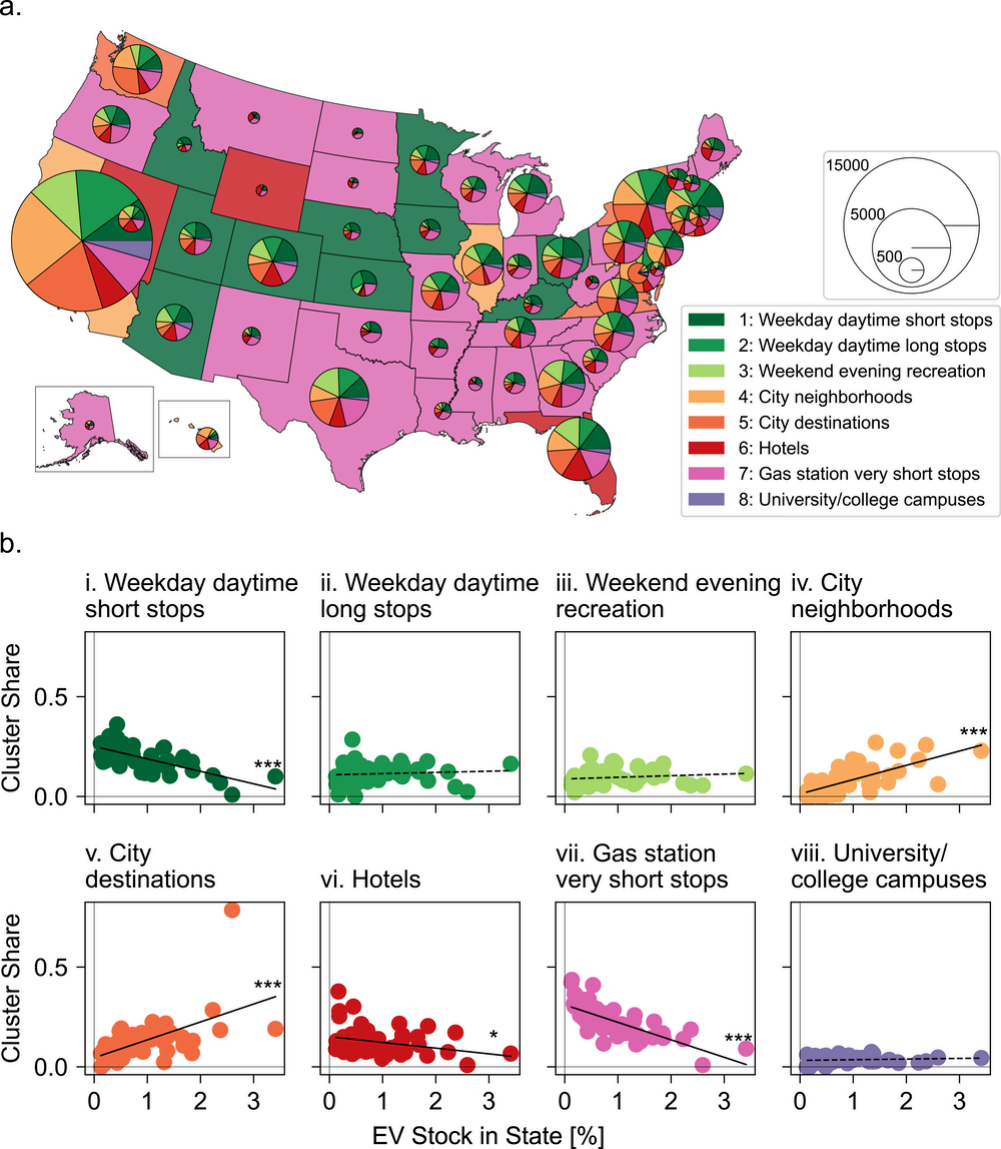
(a) The distribution of station clusters within each state. The background color of each state shows its most common cluster. The size of each pie chart scales with the number of stations in the state. (b) The share represented by each cluster (i-viii) in each state’s total set of stations vs the level of EV adoption in that state in 2023. Stars indicate the p-values for the trend lines: *** for *p* < 0.001, ** for *p* < 0.01, and * for *p* < 0.05. The absence of stars or a dashed trend line indicates *p* ≥ 0.05. We see that states with higher levels of EV adoption tend to have fewer stations in short stop clusters.

We separately estimate the neighborhood advantage for each state, as described in Section 2.2. Figures 6a and 6b summarize the results. First, for a regression of the station count against local income only, the coefficient, β_*Local Only*_, shown in Figure 6a reveals a mix of different relationships across different states: the coefficient is significantly positive for seven states, significantly negative for six states, and insignificant for 38. This replicates the mixed evidence identified by previous researchers across different city and state-specific case studies. However, when we then regress the station count against income both in the local block group and in the surrounding 10 km neighborhood, the two coefficients, β_*Local*_ and β_*Neighborhood*_, shown in Figure 6b reveal a much clearer, more consistent picture: a significantly positive neighborhood advantage for 33 states and a significantly negative local coefficient for 20 states. Only New York and South Dakota show significant effects with the opposite sign. The significance and consistency across so many states in Figure 6b.i and Figure 6b.ii suggests that the relationship with income is stronger and clearer when we look at the local and neighborhood income together.

**Figure 6:**
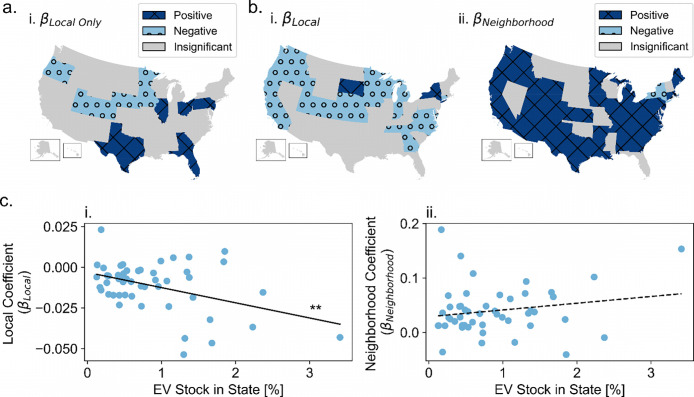
(a) Sign and significance of coefficients β_*Local Only*_ (a.i) from the regression of station count against local BG-level income only. (b) Sign and significance of coefficients β_*Local*_ (b.i) and β_*Neighborhood*_ (b.ii) from the regression of station count against local BG-level income and income in the surrounding 10km neighborhood. The regression analyses shown in this figure were all conducted separately for each state. We see that using both the local and neighborhood income resolves some of the heterogeneity in patterns across states found based on the local income only. (c) shows the relationship of the local and neighborhood coefficients, β_*Local*_ and β_*Neighborhood*_ to state-level EV adoption. The stars indicate the p-values for the trend lines: *** for *p* < 0.001, ** for *p* < 0.01, and * for *p* < 0.05. The absence of stars and the dashed trend line indicate *p* ≥ 0.05. We see that the income and neighborhood incomes have a stronger effect (coefficients larger in magnitude) in states with higher EV adoption.

We further find that these state-level effects are also related to the level of EV adoption. There is a significant relationship between the level of EV adoption in each state and the magnitude of the local coefficient, β_*Local*_, shown in Figure 6c.i (*p* < 0.01). These results suggest that the dynamics of local income play a larger role in station placement in more mature EV markets. These effects are similar for the relationship between the number of stations and a state-level count of EV-related policies (Supplementary Figure S15), and stronger than for the relationship between the number of stations and other metrics considered like the state-level Gini coefficient of income inequality or the Moran’s I metric of spatial inequality (Supplementary Figures S16 and S17).

Finally, one landmark income-based charging infrastructure policy at the US level was the 1.25 Billion USD Community Charging and Fueling Grants program introduced in the 2022 Bipartisan Infrastructure Law, which targeted funding toward disadvantaged communities with low income at the census tract level (BIL; see Supporting Information Note 1). The policy was accompanied by a label of disadvantaged communities provided by the U.S. Environmental Protection Agency which, in addition to low income, considered eight additional categories of burden criteria related to climate change impacts, energy access and affordability, health, housing, legacy pollution, transportation, water and wastewater, and workforce development in the community (see Supporting Information Note 1).

We apply the analyses developed in this paper to assess how well the EPA label of disadvantaged communities identifies locations for charging infrastructure investment and to test whether the US-wide pattern of a neighborhood advantage is also present for disadvantaged communities. Our results (in Supporting Information Note 6) show that EPA-labeled disadvantaged communities have especially low access to charging. We also find a significant positive neighborhood advantage and a significant negative effect of local income in disadvantaged communities. This suggests that the policy does identify disadvantaged areas with respect to charging access and that it could have been improved by including both local income and neighborhood income criteria.

Due to delays and long implementation timelines at the state level, almost no BIL-funded stations were included in our data sample.^44^ However, other existing and pre-existing policies could affect the results.

The state of California has had a number of smaller programs targeting support for stations to disadvantaged and low-income areas (see Supporting Information Note 1). 2.5 million USD was allocated for this purpose in 2021 as part of the state’s distribution of the Volkswagen Environmental Mitigation Trust,^61^ but that would have been enough to fund only a relatively small number of stations. Other more recent programs run by the California Electric Vehicle Infrastructure Project (CALeVIP) in 2023 and 2025 ^62,63^ and by the Los Angeles Department of Water and Power in 2023,^64^ either dedicating or prioritizing charging infrastructure funding to low-income and disadvantaged communities, were too late to affect the results in this study. Programs in the states of New York and Colorado were similarly too small and too late to have affected the results (see Supporting Information Note 1 for more details). Future research could explore how these newer policies will affect the distribution of chargers in these areas.

Many policies have not limited their funding to low-income areas but have tried to improve network coverage across the United States. For example, starting in 2017 Volkswagen invested 2 billion USD in the Electrify America fast-charging network as part of its settlement with the U.S. government after the diesel emissions scandal.^65^ The U.S. federal government provided long-term support for businesses installing level 2 charging through the Alternative Refueling Infrastructure tax credit between 2005 and 2022,^66^ and moved in 2015 to define a map of alternative fuel corridors (AFCs) to identify key highway routes to cover in a national charging network.^67^

These policies likely contributed to the number of highway-side fast-charging stations we observe in our clustering results. These have distinct mobility patterns with very short stops and are located both in low- and high-income areas, though they make up a relatively higher proportion of stations in low-income counties (Figure 4a–c). Notably, they do not have a significant neighborhood advantage (Figure 4d), which is consistent with the idea that their placement may have been driven by the need for even network coverage rather than considerations of local income patterns.

## 4 Discussion

First, our analysis identified a robust neighborhood advantage, where charging station deployment in low-income communities is facilitated by the presence of surrounding high-income communities. Though local income is used by some existing policies as the only income-based criterion, we find that local income alone can have a positive or negative relationship with the number of public charging stations across different US states; a result matching the mixed evidence found in previous literature on city- or state-specific case studies.^29–34^ Local income alone could omit important spillovers between neighborhoods enabled by the mobility of drivers and co-location of stations with different POIs. Some low- and mid-income communities will benefit from private companies building stations to target demand from nearby high income areas. This is different from other technologies which do not benefit from mobility, such as rooftop solar. When we additionally consider the neighborhood income, we find a more consistent relationship with charging station deployment, helping untangle the heterogeneity found by other studies.

Future policies based on income should therefore consider both the local and neighborhood income levels. Policy designs that could be considered may include: a maximum limit to qualify for funding could be applied to both the local and neighborhood income; the neighborhood income level could be included as part of a multicomponent scoring criteria in funding applications; or the number of stations already in the local community could be used in addition to income to help policymakers identify communities with the least charging access. Future research should explore the implications of different policy implementations.

Second, charging stations built in low-income areas seem to be less accessible to low-income drivers than to high-income drivers. Policy makers should move beyond targeting just the count of charging stations and also consider the charging station types and locations. Today, areas with high EV adoption show a wide variety of different charging station types, suggesting that a similar mix should be targeted for lower-income areas to serve the needs of local drivers.

Finally, our findings show that these effects are stronger in states with higher EV adoption and policy support. As the neighborhood advantage plays a larger role in more mature EV markets, it is increasingly important for policymakers to take this into account.

In summary, this study uncovers the neighborhood advantage and its importance for the design of effective income-based policies for charging infrastructure. Future research should aim to identify which communities currently do not benefit from network effects. Additional data including mobility data divided by income level and driver-specific charging histories could further reveal who is using which station types to extend our insights on the accessibility of charging stations. Changes to mobility patterns beyond October 2022 could also affect the results if they change charging station companies’ investment decisions. These could include more charging for commuters following “return-to-office” postpandemic policies or more charging in neighborhoods with growing EV adoption. Future research should also investigate how seasonality affects charging station placement, considering mobility patterns related to peak vacation travel periods or affected by changing weather. Finally, more granular data on EV adoption would make it possible to explicitly analyze the effectiveness of policies targeting low- and mid-income areas for EV adoption.

To conclude, as policymakers look to support the next phases of EV adoption, understanding the role of income in EV charging station placement is critical. Policymakers in the US and around the world can leverage our findings to design better income-based policies and better target their support to where it is needed most. A rich array of accessible, affordable, and convenient charging options in all neighborhoods is an important step to unlocking truly widespread electrification.

## Supplementary Material



## Data Availability

The data generated in this study needed to reproduce the main figures is available at https://data.mendeley.com/datasets/v22k6k63sx/1. The Safegraph mobility patterns data was obtained from Dewey Data and was confidentially provided under an NDA. Raw data sources for the other data used in this study (i.e., charging station locations and technical characteristics, census data) are publicly available; the sources are described in the Methods section. All code used in this study is available at https://github.com/SiobhanPowell/income-based-charging-station-policies.

## Notes

Additional information, figures, and tables including background on relevant policies, robustness checks of the main results and methods choices, supplementary analysis of a particular policy case, and further details on the data sets and data processing (PDF)
