# Supplementary material for: Mobility and Income: Policy Insights to Support Public Electric Vehicle Charging Access
Source: Environ Sci Technol. 2026 Jun 29;60(27):19012–24. doi: 10.1021/acs.est.6c02101 (PMC13374092; doi:10.1021/acs.est.6c02101)
Supplement: Supplementary file 1 [file es6c02101_si_001.pdf]

Supplementary Information: Mobility and Income: Policy Insights to Support Public Electric Vehicle Charging Access

Siobhan Powell[1*†] and Marie-Louise Arlt[2,3,4*†]

[1]Group for Sustainability and Technology, Swiss Federal Institute of Technology Zurich (ETH Zurich), Weinbergstrasse 56/58, Zurich, 8092, Zurich, Switzerland.
[2]Department of Law, Economics, and Business Administration, University of Bayreuth, Universitätsstrasse 30, 95447 Bayreuth, Germany.
[3]Bavarian Center for Battery Technology, University of Bayreuth, Weiherstrasse 26, 95448 Bayreuth, Germany.
[4]ifo Institute, Poschingerstrasse 5, 81679 Munich, Germany.

*Corresponding author(s). E-mail(s): spowell@ethz.ch; arlt@uni-bayreuth.de;
†S.P. and M.L.A. contributed equally to this paper.

8 supplementary notes, 40 pages, 36 figures, 11 tables.

Supplementary Note 1: Income-Based Policies for Charging Infrastructure

Multiple recent policies in the US have explicitly included subsidies and support for new charging stations to low-income residents or areas.

The 2022 Inflation Reduction Act included support for charging infrastructure, with tax credits for up to 30% of costs for new charging stations (capped). The Inflation Reduction Act guidebook states that the 30% tax credit is only available to chargers in low-income and rural communities (p. 53) [1].

An even larger such program was included in the 2022 Bipartisan Infrastructure Law (BIL) [2]. The BIL allocated 5 Billion USD toward the National Electric Vehicle Infrastructure Formula Program (NEVI) Formula program, building a national network of fast chargers along highway corridors, and 2.5 Billion USD toward the Charging and Fueling Infrastructure Discretionary Grant program. This 2.5 Billion USD was split between Corridor Grants (1.25 Billion USD) and Community Charging and Fueling Grants (1.25 Billion USD). The Infrastructure Law guidebook states the goal that "This investment will make electric vehicles accessible to all Americans, ... and ensure a convenient, reliable, affordable, and equitable charging experience for all users" (p. 136). Recent research on the NEVI program found it would substantially improve continuous charging coverage in the US, but still leave some gaps in rural counties [3].

The Community Charging and Fueling Grants explicitly target funding for slow public chargers toward low-income and disadvantaged communities. The guidebook states that Community Grants will be prioritized for "rural areas, low-and-moderate-income neighborhoods, and communities with low ratios of private parking, or high ratios of multiunit dwellings" (p. 142) [2]. The exact definition of these terms is left to the states, but can be facilitated by use of the US Environmental Protection Agency (EPA) labels of disadvantaged communities on a census tract level given by the Climate and Economic Justice Screening Tool [4]. With the CEJST, communities in the US (excluding US Territories) are labeled disadvantaged if they (A) are on Tribal lands or (B) are low income (in the 65th percentile or higher for percentage of population in households with income $\leq$ twice the federal poverty level) and in the 90th percentile of at least one other burden criterion. Burden criteria are grouped into eight categories: climate change, energy, health, housing, legacy pollution, transportation, water and wastewater, and workforce development. CTs that are completely surrounded by disadvantaged CTs are also considered disadvantaged if they also fall below the 50th income percentile [4].

In California, the California Electric Vehicle Infrastructure Project (CALeVIP) prioritizes funding for disadvantaged communities in its Fast Charge California Project [5]. Previous CALeVIP grants exclusively targeted low-income and disadvantaged communities [6]. In 2021, 2.5 million USD of funding from the Volkswagen Environmental Mitigation Trust in California was made available to support new charging stations in disadvantaged and low-income communities [7].

The city of Los Angeles, California has a number of programs targeting charging stations to disadvantaged communities. Through the "Powered by Equity" program,

the local utility, Los Angeles Department of Water and Power (LADWP) is itself installing charging stations in "underserved communities" [8]. LADWP also offers higher rebates for private chargers to low-income customers [9].

Some utilities outside of California have also recently introduced similar programs. For example, Black Hills Energy in Colorado offers larger rebates for low-income residents installing EV chargers [10]. The Charge Ready NY program run by the New York State Energy Research and Development Authority (NYSERDA) also provides larger subsidies for public and private commercial chargers in disadvantaged communities [11].

Outside the US, policy support so far has typically focused on broad coverage. Some target multi-unit dwellings and residents without off-street parking, but we could not find any with an explicit tie to income.

Supplementary Note 2: Additional Information for Figure 1

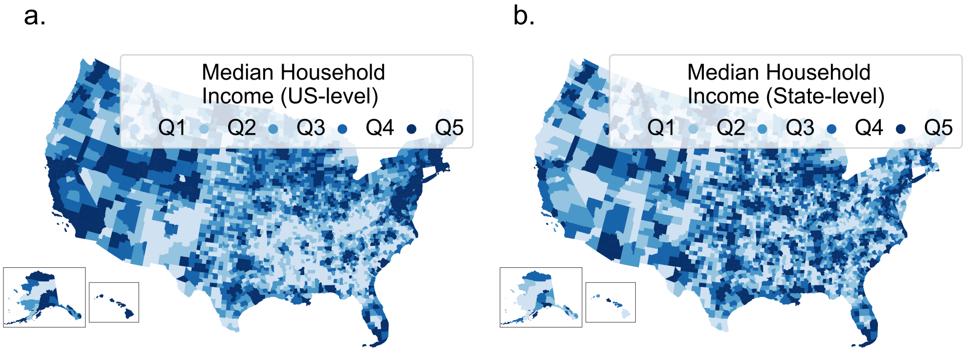

Fig. S1: Median household income for each county using quintiles calculated at the US level (a) and state level (b).

We use both US-level and state-level income quintiles in our analysis. Figure S1 illustrates a comparison between these two metrics at the county level. When quintiles are calculated at the state level, we see both high- and low-income counties in each state on the map.

Figure S2 shows the total number of stations by BG and county income quintile, when the quintiles are calculated at the US level (a) or state level (b). The total aggregated by county, census tract (a geographic unit between block group and county), or block group is shown in Figure S3 for the US-level quintiles and in Figure S4 for the state-level quintiles.

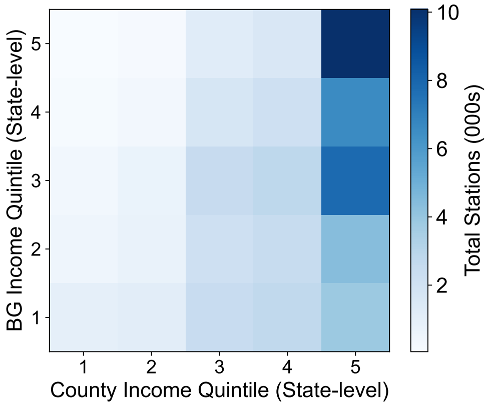

Fig. S2: Comparison of station placement using US-level and state-level income quintiles.

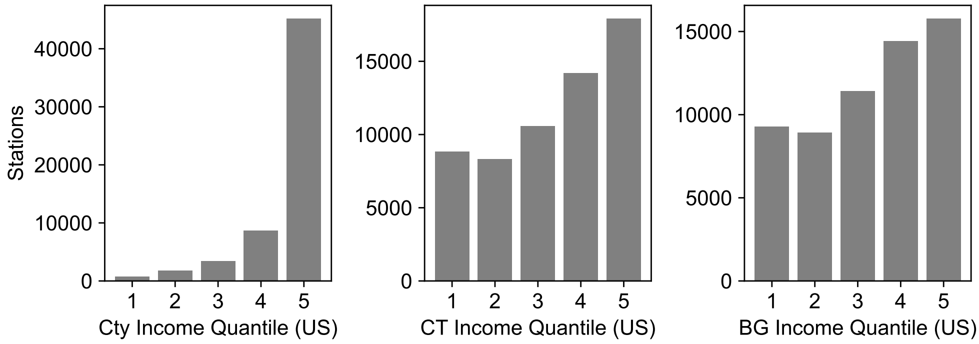

Fig. S3: Total number of stations aggregated by county (Cty), census tract (CT), and block group (BG) income quintile, with quintiles calculated at the US level.

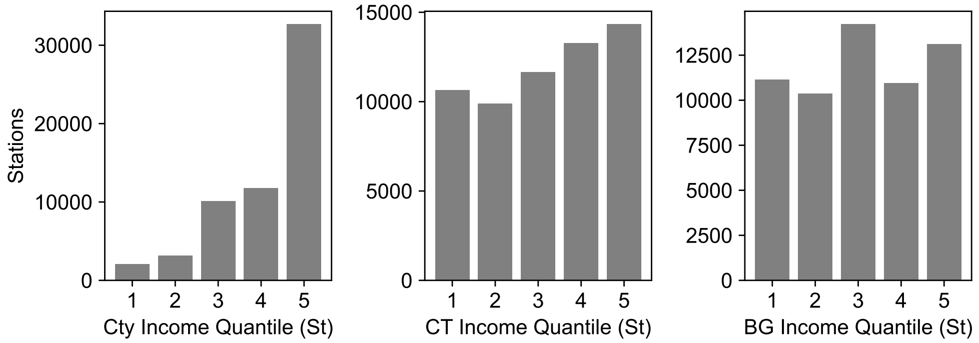

Fig. S4: Total number of stations aggregated by county (Ct), census tract (CT), and block group (BG) income quintile, with quintiles calculated at the state level.

Supplementary Note 3: Regression Analysis - Robustness Checks

Table S1: Extended version of Table 1.

	Dependent variable: Number of stations				
	(1)	(2)	(3)	(4)	(5)
BG median household income [10k USD]	0.012***	0.003***	-0.012***	-0.003***	-0.012***
	(0.001)	(0.001)	(0.001)	(0.001)	(0.001)
Average neighbouring income [10k USD], BG/10km			0.056***		0.056***
			(0.002)		(0.002)
Average neighbouring income [10k USD], BG/50km				0.054***	
				(0.003)	
Distance to nearest highway [100 km]					-0.017
					(0.013)
Unit of observation	BG	BG	BG	BG	BG
State FE	No	Yes	Yes	Yes	Yes
Observations	239780	239780	239766	239776	239766
R^2	0.001	0.010	0.013	0.012	0.013
Adjusted R^2	0.001	0.010	0.013	0.012	0.013
Residual Std. Error	1.650	1.643	1.640	1.641	1.640
F Statistic	210.165***	48.536***	62.087***	55.224***	60.946***

Note: *p<0.05; **p<0.01; ***p<0.001

Table S2: Extension of Table 1 with analysis at the Census Tract (CT) level.

	Dependent variable: Number of stations				
	(1)	(2)	(3)	(4)	(5)
CT median household income [10kUSD]	0.055***	0.026***	-0.025***	0.004	-0.025***
	(0.003)	(0.003)	(0.004)	(0.003)	(0.004)
Average neighbouring income [10kUSD],			0.157***		0.157***
BG/10km			(0.007)		(0.007)
Average neighbouring income [10kUSD],				0.147***	
BG/50km				(0.009)	
Distance to nearest highway [100km]					-0.025
					(0.041)
Unit of observation	CT	CT	CT	CT	CT
State FE	No	Yes	Yes	Yes	Yes
Observations	84414	84414	84404	84411	84404
R^2	0.004	0.025	0.031	0.028	0.031
Adjusted R^2	0.004	0.024	0.030	0.027	0.030
Residual Std. Error	3.042	3.011	3.002	3.006	3.002
F Statistic	336.384***	42.111***	51.698***	46.776***	50.729***

Note: *p<0.05; **p<0.01; ***p<0.001

We compiled the CT-level dataset by aggregating the BG-level dataset by FIPS code and re-computing each column. For additive columns, such as the total population in a CT, we add all values of the BGs contained in the CT. For average columns, such as median household income, we compute the population-weighted average of values of the BGs contained in the CT.

Table S3: Extension of Table 1 with alternative specifications. Model 1 is the base specification. Model 2 is the base specification, weighted by population within the BG. Model 3 is run for only BG which have stations. Model 4 is a Poisson and Model 5 a Negative Binomial model. Model 6 is run as a Logit regression on the fact of whether a BG has any stations.

	Dependent variable					
	# stations					*Has stations*
	(1)	(2)	(3)	(4)	(5)	(6)
BG median household income	-0.012***	-0.013***	-0.016	-0.038***	-0.033***	-0.034***
[$10kUSD$]	(0.001)	(0.001)	(0.009)	(0.001)	(0.001)	(0.002)
Average neighbouring income	0.056***	0.059***	0.177***	0.165***	0.159***	0.123***
[$10kUSD$], $BG/10km$	(0.002)	(0.002)	(0.016)	(0.002)	(0.003)	(0.004)
Unit of observation	BG	BG	BG	BG	BG	BG
State FE	Yes	Yes	Yes	Yes	Yes	Yes
Observations	239766	239766	24660	239766	239766	239766
R^2	0.013	0.015	0.031			
Adjusted R^2	0.013	0.015	0.029			
Pseudo R^2						0.032
Residual Std. Error	1.640	1.640	4.541			
F Statistic	62.087***	70.866***	14.939***			

Note: *p<0.05; **p<0.01; ***p<0.001

Table S4: Extension of Table 1 with additional covariates. Model 4 includes only urban BGs and model 5 includes only rural BGs. Urban BGs include BGs with population density above median, rural BGs below median population density.

	Dependent variable: # stations				
	(1)	(2)	(3)	(4)	(5)
BG median household income [10kUSD]	-0.012***	-0.011***	-0.013***	-0.005***	-0.034***
	(0.001)	(0.001)	(0.001)	(0.001)	(0.002)
Average neighbouring income [10kUSD], BG/10km	0.056***	0.054***	0.058***	0.037***	0.108***
	(0.002)	(0.002)	(0.002)	(0.002)	(0.004)
Has highway passing through		0.406***			
		(0.008)			
Population density			-8.352***		
			(0.617)		
Unit of observation	BG	BG	BG	BG	BG
State FE	Yes	Yes	Yes	Yes	Yes
Observations	239766	239766	239167	119589	119578
R^2	0.013	0.023	0.014	0.012	0.027
Adjusted R^2	0.013	0.022	0.014	0.012	0.026
Residual Std. Error	1.640	1.632	1.642	1.375	1.860
F Statistic	62.087***	104.694***	64.323***	27.782***	62.813***

Note: *p<0.05; **p<0.01; ***p<0.001

Table S5: Analysis of Table 1 repeated using 2014 ACS median household income data, to limit the reverse effect of charging stations on economic outcomes in the analysis. 92.1% of charging stations in the dataset have been built in 2015 or later. 2014 ACS median income data is sourced from [12] and was matched to 2020 BG definitions based on [13].

	Dependent variable: Number of stations		
	(1)	(2)	(3)
2014 BG median household income [10kUSD]	0.001	-0.017***	-0.006***
	(0.001)	(0.001)	(0.001)
2014 Average neighbouring income [10kUSD], BG/10km		0.064***	
		(0.003)	
2014 Average neighbouring income [10kUSD], BG/50km			0.066***
			(0.004)
Observations	237315	237315	237315
R^2	0.010	0.013	0.011
Adjusted R^2	0.010	0.012	0.011
Residual Std. Error	1.599	1.597	1.598
F Statistic	46.951***	58.112***	52.607***

Note: *p<0.05; **p<0.01; ***p<0.001

Supplementary Note 4: POI and Clustering Details

We map the POIs in the SafeGraph data to 10 higher level categories: commercial, manufacturing, recreation, education/child care, transit, gas station, medical, office, hotel, and restaurants and bars. Note here "manufacturing" refers to POIs in a range of industrial, manufacturing, and infrastructure sectors. The mapping is given in Supplementary Table S10.

Figure S5 shows that the number of POIs decreases on average with increasing BG-level within-county relative income for all types of POIs. The shape and distribution over income levels, however, varies between the POI types. For example, Education/Child Care POIs are more evenly distributed than Transit or Restaurants and Bars. This motivates the use of different POIs in the clustering.

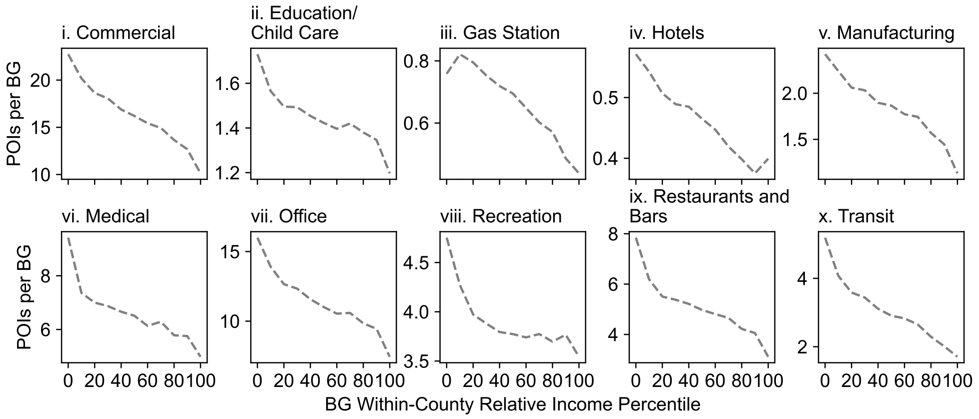

Fig. S5: Average number of POIs of each type by within-county BG-level income percentile.

There are 234 POIs within a 500m radius of the average charging station (Figure S6). Commercial POIs are the most common category among nearby POIs (Figure S7).

Figure S8 illustrates the cluster centers against all clustering variables. Table S6 discusses the mean characteristics of stations in each cluster, based on Figure S8. In Figure S8 we separate and group the 52 input features into six subplots for easier interpretation.

The travel pattern data includes a metric of visitors' average distance from home when they visit a given POI. We calculate the average distance from home for each station location by taking the mean of this metric over all POIs within 500 meters. Figure S9 shows the average value of this metric for station locations in each of the eight clusters.

Figure S10 repeats Figure 4 from the main manuscript with annotated values. Figure S11, S12, and S13 show the distribution of stations in each cluster by BG and County income quintile.

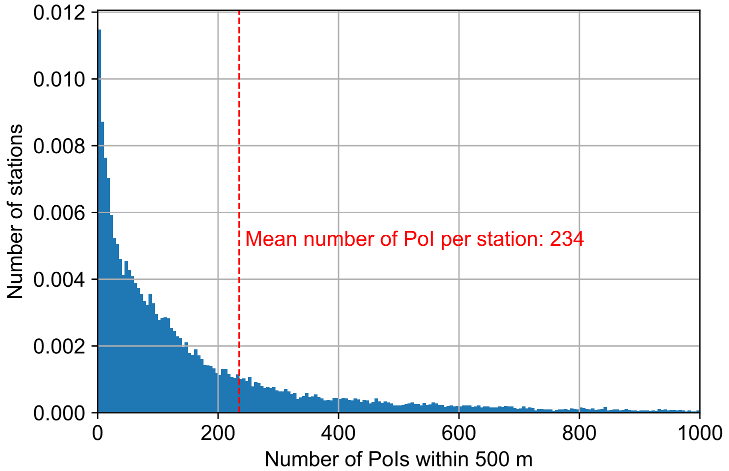

Fig. S6: Distribution of number of PoIs within 500m by station.

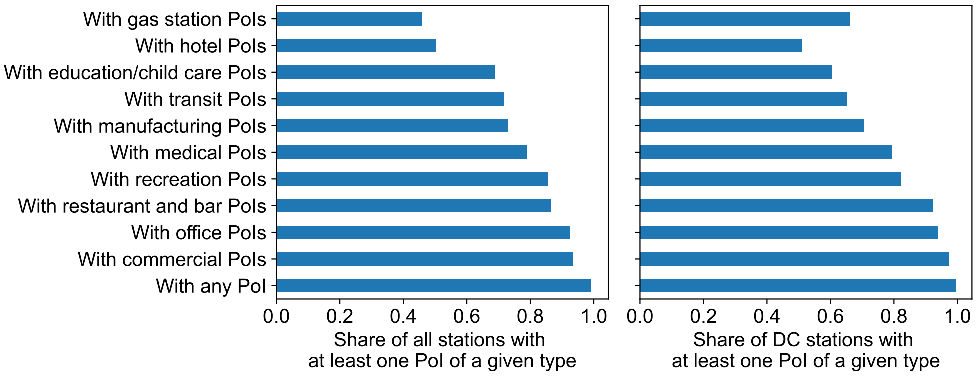

Fig. S7: Share of stations with at least one PoI of a given type within 500m.

Figure S14 shows the distribution of clusters among stations within each state, plus the District of Columbia.

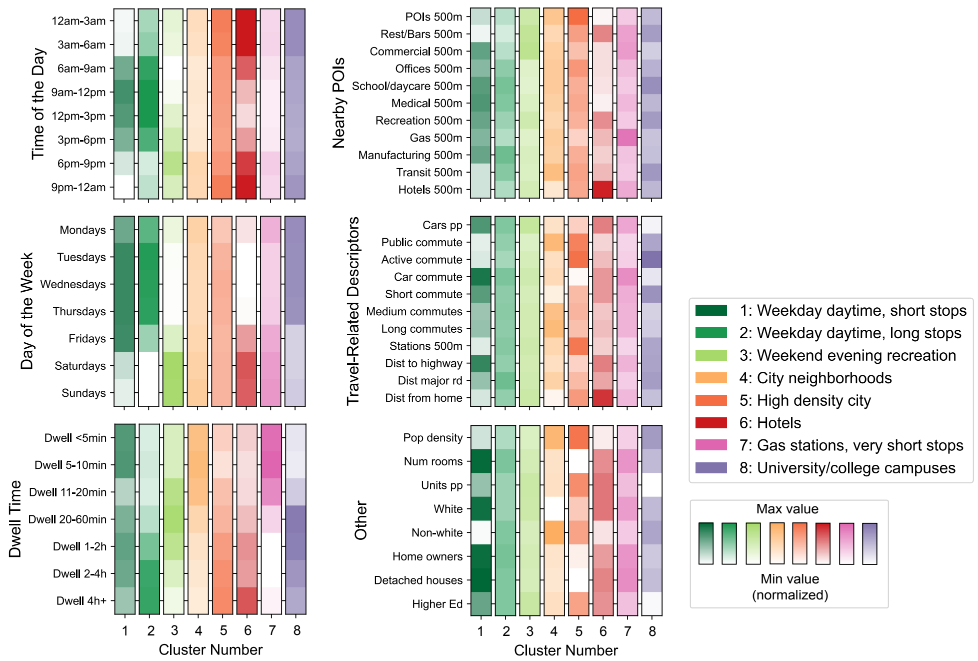

Fig. S8: Heatmap of cluster centers showing how high or low the average station from each cluster places in each of the clustering variables.

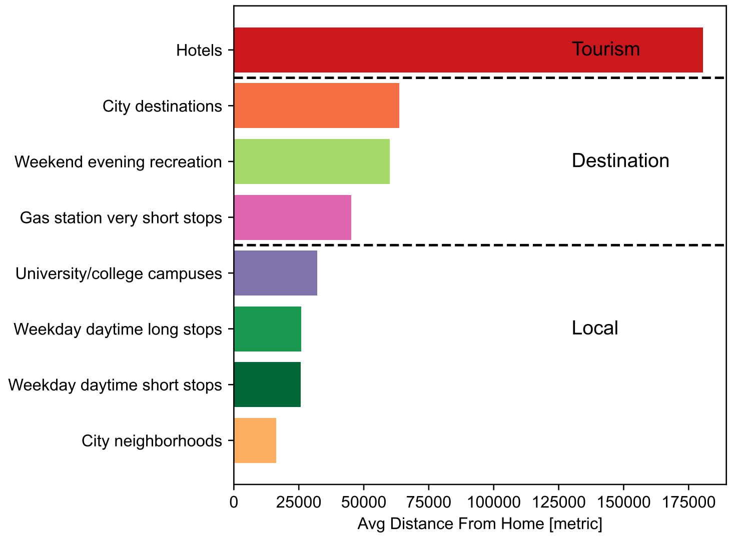

Fig. S9: Mean distance from home by cluster. The labels "Tourism", "Destination", and "Local" are added to describe different groups of the clusters based on this metric.

Number	Cluster Name	Description
1	Weekday daytime short stops	Stations in this cluster are near POIs visited most frequently on weekdays during the day, including both workplace and commercial POIs, many with very short typical dwell times.
2	Weekday daytime long stops	Stations in this cluster are near POIs visited most frequently on weekdays during the day, but with long typical dwell times, suggesting these are co-located with workplaces or schools.
3	Weekend evening recreation	PoIs in the neighborhood of this type of station are mostly visited on Fridays through Sundays between 6pm and 9 pm. Restaurants and other commercial PoI are over-represented. Usual dwell times are between 11 minutes and two hours.
4	City neighborhoods	City stations are located in BGs with high population density and a large fraction of public transit or active commuters. This cluster is differentiated from the other city cluster by the extremely low average distance from home for visitors to nearby POIs, suggesting these are very local destinations.
5	City destinations	City stations are located in BGs with high population density and a large fraction of public transit or active commuters. In this cluster, within 500m, stations are surrounded by a very high number of POIs and other charging stations.
6	Hotels	Stations of this cluster type are primarily co-located with hotels, and are visited on weekends during the night for dwell times of four hours or more. Visitors are traveling a rather long way from home.
7	Gas stations very short stops	Locations in this cluster are located near gas station and comericial POIs with very short dwell times, almost exclusively less than 20 minutes duration.
8	University/college campuses	This cluster of locations have a quite unique mobility pattern, very popular Monday through Friday and overnight, with a relatively high concentration of school, restaurant or bar, recreation, and transit POIs. Looking carefully at individual locations from this cluster, all were located on or near college or university campuses. The concentration of dwell times between 20 minutes and 4 hours could correspond to students attending lectures or other campus activities.

Table S6: More detailed discussion of cluster descriptions.

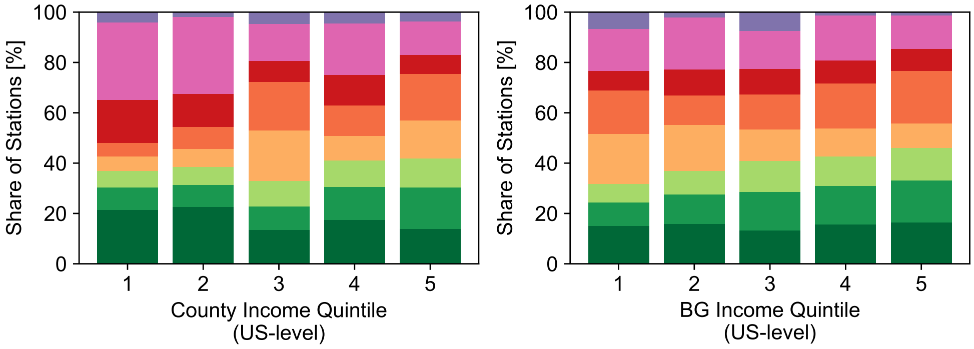

Fig. S10: Version of Figure 4 with annotated values.

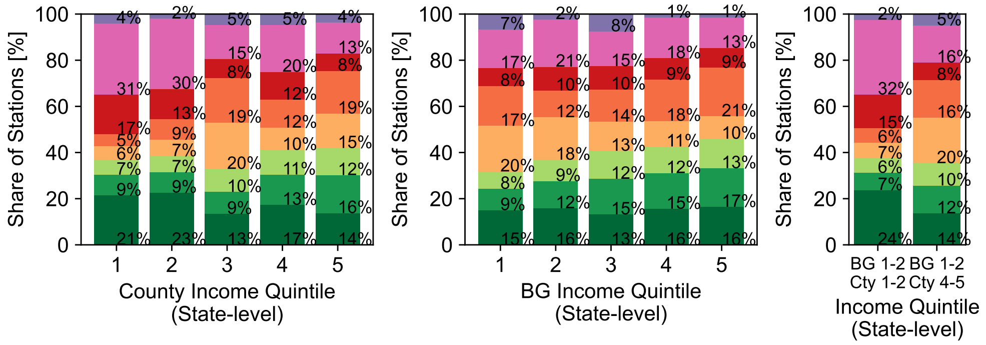

Fig. S11: The share of all stations over the eight clusters for counties and BGs in each income quintile, as calculated at the US level.

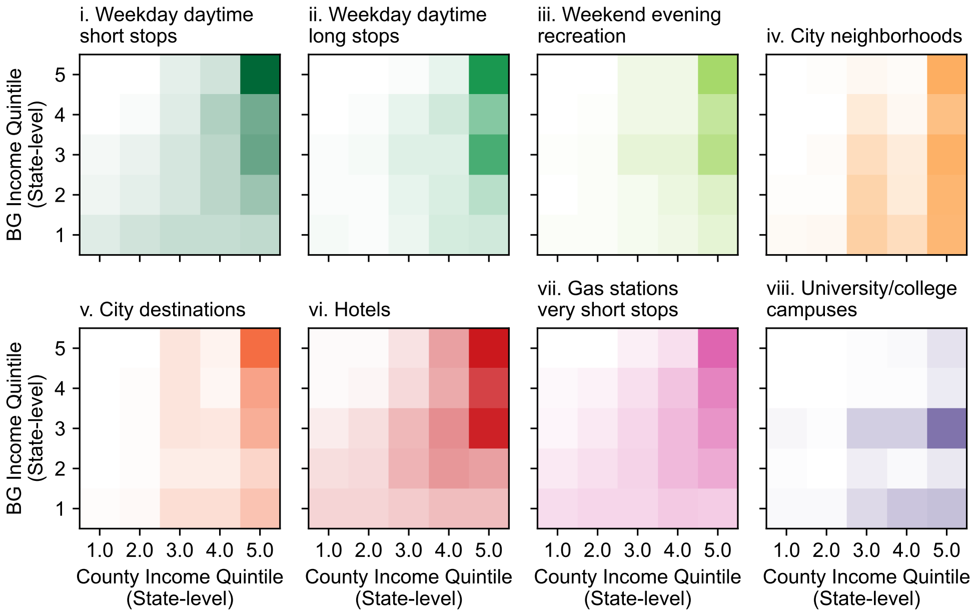

Fig. S12: Heatmap of station counts for each cluster by BG-level and county-level income quintile, using state-level income quintiles.

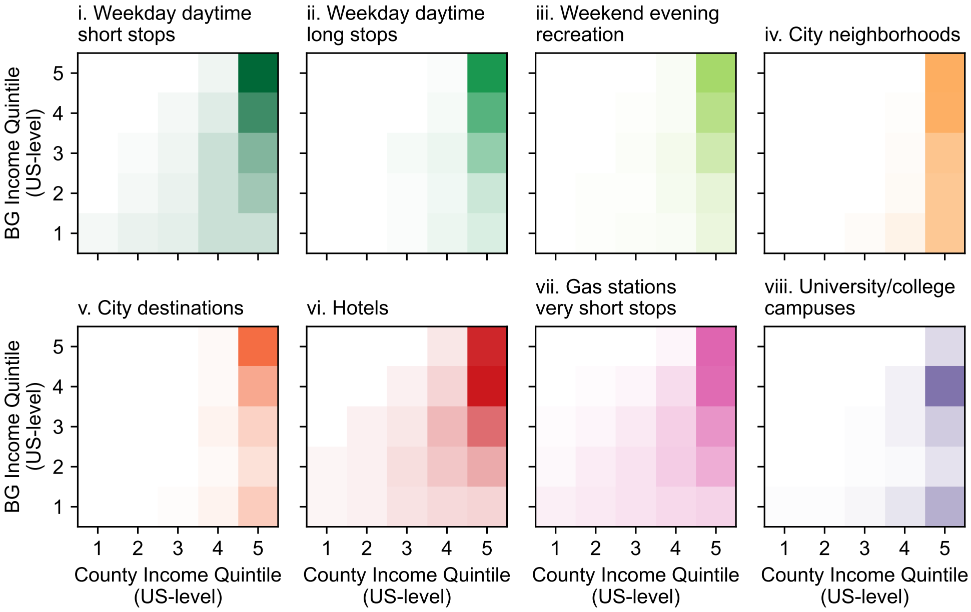

Fig. S13: Heatmap of station counts for each cluster by BG-level and county-level income quintile, using US-level income quintiles.

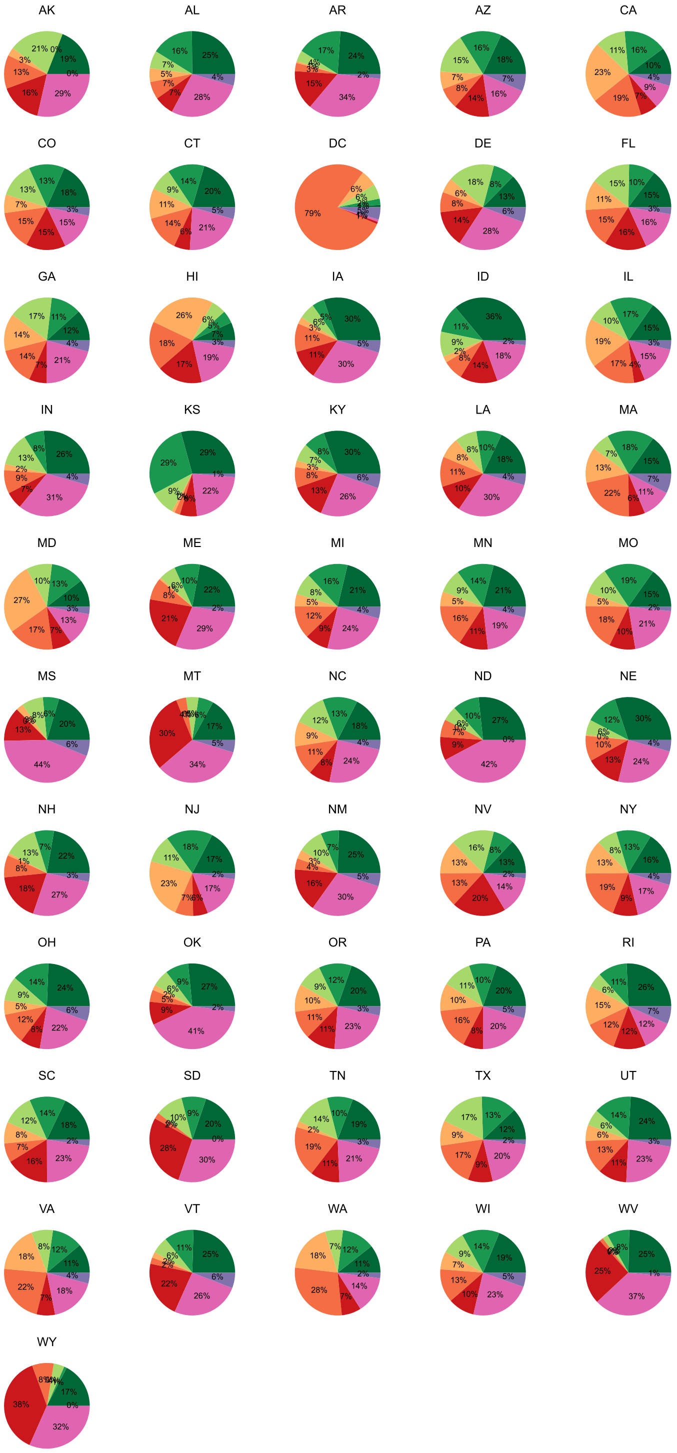

Fig. S14: For each state, the share of stations that fall in each of the eight clusters.

Supplementary Note 5: Relationship between Station Types, POIs, Gini Coefficient, and EV Stock

Figure S15 shows the relationship at the state level between the count of EV-related policies and the local or neighborhood advantage. We access data on state-level policies related to EVs from the AFDC [14] and filter for those that were introduced by 2022 and not yet expired in 2023. The count of policies and level of EV adoption in each state are correlated (Pearson correlation coefficient of 0.81).

We find there is a significant relationship with the number of EV-related policies in each state (see Methods), both for the local and neighborhood advantage (Figure S15, p<0.01 for both).

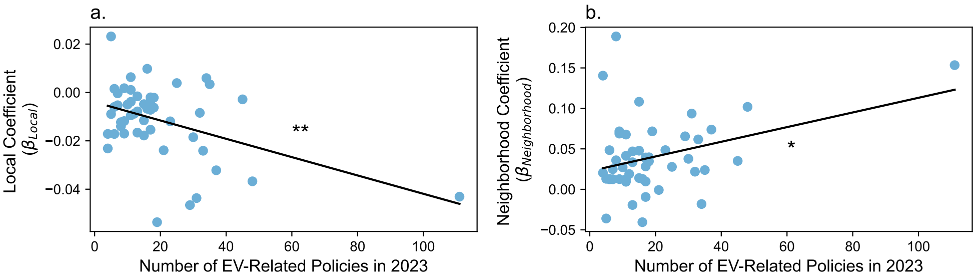

Fig. S15: Relationship between the state-level count of EV-related policies and the local (i) and neighborhood (ii) advantage coefficients. Stars indicate the p-values: *** for p<0.001, ** for p<0.01, and * for p<0.05. The absence of stars or a dashed trend line indicates p≥0.05.

Figures S16 and S17 show the relationship at the state level between the Gini coefficient and Moran's I coefficient, two measures of inequality, and the local or neighborhood advantage and the distribution of stations by cluster.

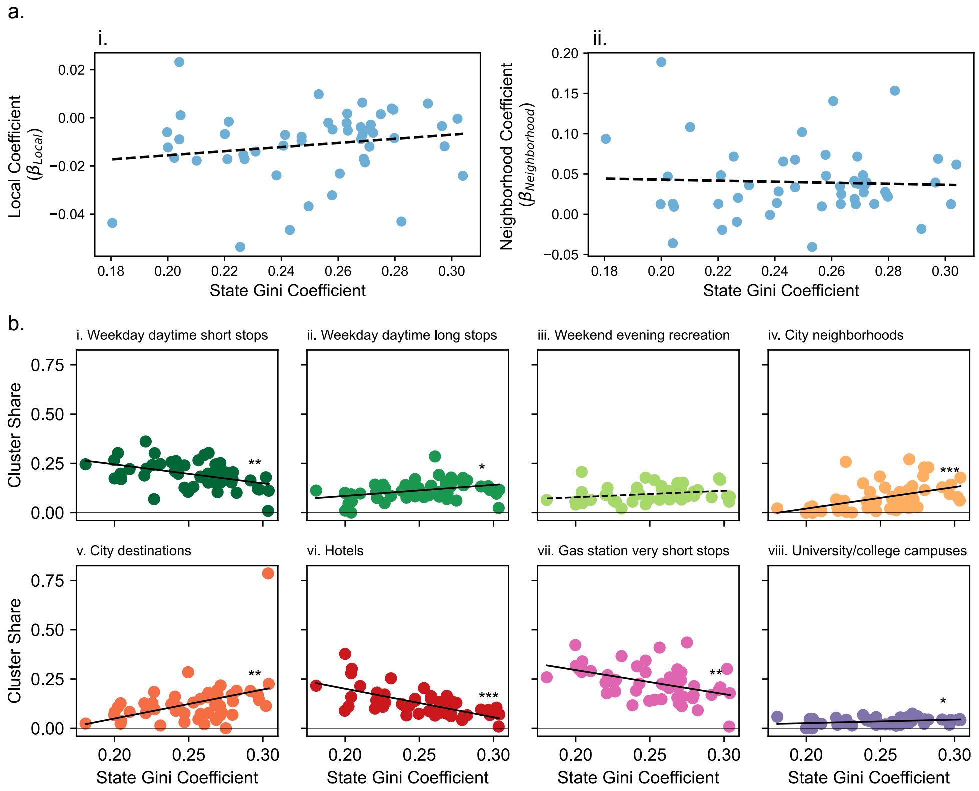

Fig. S16: Relationship between state Gini coefficients (calculated with BG-level data) and (a) local and neighborhood advantage, or (b) share of stations in each cluster. Stars indicate the p-values: *** for p<0.001, ** for p<0.01, and * for p<0.05. The absence of stars or a dashed trend line indicates p≥0.05.

Supplementary Note 6: Analysis with BIL DAC Criteria

The share of each county's population that falls in an EPA-labeled disadvantaged community (DAC) is shown in Figure S18. This labeling using the CEJST is described in detail in Supplementary Note 1. Compared with Figure 1, we can see that many of the counties with the lowest population share in DACs are also the counties with the lowest access to public chargers. The distribution of DACs is also different from the distribution of low-income communities, reflecting the many other criteria of disadvantage in the label and the effect of aggregating from the census tract to county level.

We find there are 11,679 public charging stations in census tracts (CTs) labeled as DACs, compared to 31,795 in non-DAC CTs: a factor of 2.7×. Figure S19 shows the normalized count per CT. Surprisingly, we see that the small number of CTs triggering

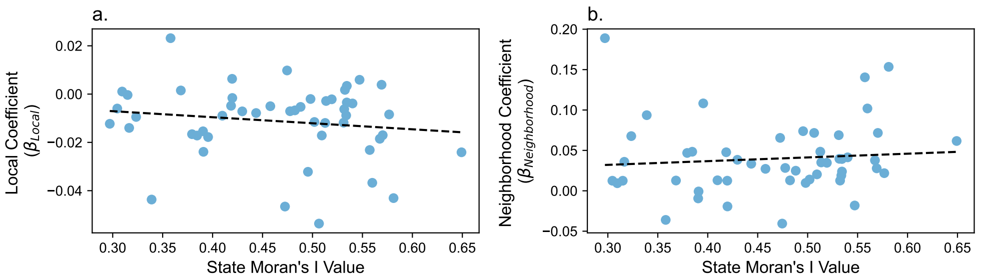

Fig. S17: Relationship between state Moran's I coefficient (calculated with BG-level data) and local and neighborhood advantage. Stars indicate the p-values: *** for $p<0.001$, ** for $p<0.01$, and * for $p<0.05$. The absence of stars or a dashed trend line indicates $p\geq0.05$.

4 or more DAC criteria show higher numbers of charging stations than those triggering fewer criteria.

If a census tract does not surpass any of these thresholds to be labeled disadvantaged, but every one of its neighboring census tracts does, and it satisfies a looser low-income criteria, then it is also labeled disadvantaged by the "All Neighbors" criterion. We observe that census tracts satisfying the "All Neighbors" criterion have a lower number of public EV charging stations than DACs that qualified by any other criteria. Further, the higher the share of its neighbors that are labeled disadvantaged, the fewer charging stations are located in the census tract (Figure S19c and d). This is consistent with our finding that low-income BGs within low-income counties have the fewest stations, especially compared to low-income BGs within high-income counties.

Table S7 shows that the main observation in the paper, of a negative local income effect and positive neighborhood advantage, are unchanged by including the DAC label in the regression. Further, models 5 and 6 show that this effect is different for the DAC label alone: both the local DAC label and share of the neighborhood in a DAC have a significant negative association with the number of public charging stations. Table S8 shows that the negative local income effect and positive neighborhood advantage are also significant when we split BGs into DACs (models 1 and 2) and non-DACs (models 3 and 4).

Figure S20 shows the share of stations in each cluster in DAC and non-DAC CTs. Different from the income-only criteria used in the main analysis of the paper, here we see that the City Neighborhoods cluster is larger in DACs than non-DACs, reflecting that many inner city areas are affected by the other burden criteria.

Table S7: Regression analysis at the block group (BG) level of the relationship between the number of public charging stations, local and neighboring income (as in Table 1), whether the BG is in an EPA-labeled DAC, and the share of the population within a 10 or 50km radius in an EPA-labeled DAC.

	Dependent variable: Number of stations					
	(1)	(2)	(3)	(4)	(5)	(6)
BG median household income	0.008***	-0.003**	-0.015***	-0.009***		
[$10kUSD$]	(0.001)	(0.001)	(0.001)	(0.001)		
Average neighboring income			0.053***			
[$10kUSD$], BG/10km			(0.002)			
Average neighboring income				0.052***		
[$10kUSD$], BG/50km				(0.003)		
BG in DAC	-0.088***	-0.111***	-0.073***	-0.103***	-0.065***	-0.071***
	(0.008)	(0.008)	(0.008)	(0.008)	(0.009)	(0.008)
DAC share, BG/10km					-0.135***	
					(0.017)	
DAC share, BG/50km						-0.298***
						(0.020)
Unit of observation	BG	BG	BG	BG	BG	BG
State FE	No	Yes	Yes	Yes	Yes	Yes
Observations	239780	239780	239766	239776	239776	239779
R^2	0.001	0.011	0.014	0.013	0.011	0.012
Adjusted R^2	0.001	0.011	0.013	0.012	0.011	0.012
Residual Std. Error	1.650	1.642	1.640	1.641	1.642	1.641
F Statistic	167.746***	51.256***	62.451***	57.317***	52.347***	55.247***
Note:						*p<0.05; **p<0.01; ***p<0.001

Table S8: Regression analysis BG level repeated for BGs labeled DACs (models 1 and 2) and not labeled DACs (models 3 and 4) separately.

	Dependent variable: Number of stations			
	(1)	(2)	(3)	(4)
BG median household income [10k USD]	-0.008**	-0.003	-0.017***	-0.012***
	(0.003)	(0.003)	(0.001)	(0.001)
Average neighboring income [10k USD], BG/10km	0.052***		0.052***	
	(0.004)		(0.003)	
Average neighboring income [10k USD], BG/50km		0.030***		0.065***
		(0.004)		(0.004)
Observations	83508	83511	156258	156265
R^2	0.010	0.009	0.014	0.014
Adjusted R^2	0.009	0.008	0.014	0.013
Residual Std. Error	1.508	1.509	1.706	1.706
F Statistic	16.193***	13.808***	43.892***	41.864***
Note:				*p<0.05; **p<0.01; ***p<0.001

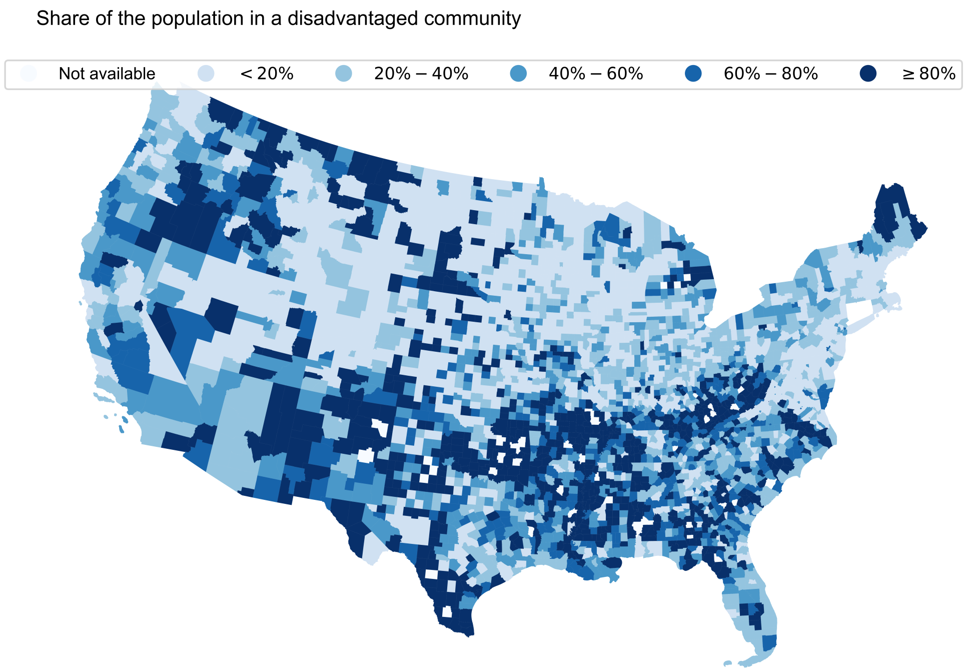

Fig. S18: County-level map showing the percentage of the population living in an EPA-designated DAC CT.

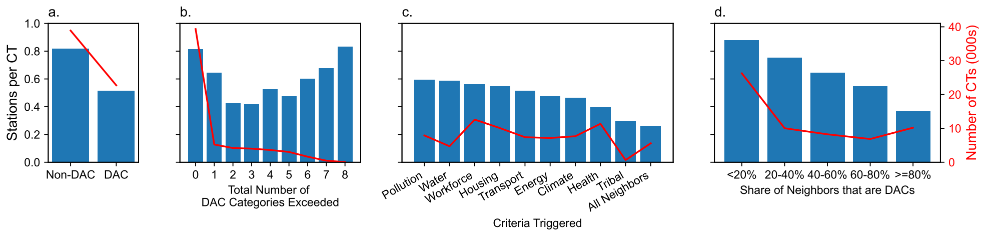

Fig. S19: The number of stations located in census tracts (CT) as aggregated by (a) DAC label, (b) the number of criteria triggered in the EPA's DAC labeling, (c) which criteria are triggered, and (d) the share of neighboring CTs labeled disadvantaged by the EPA labeling.

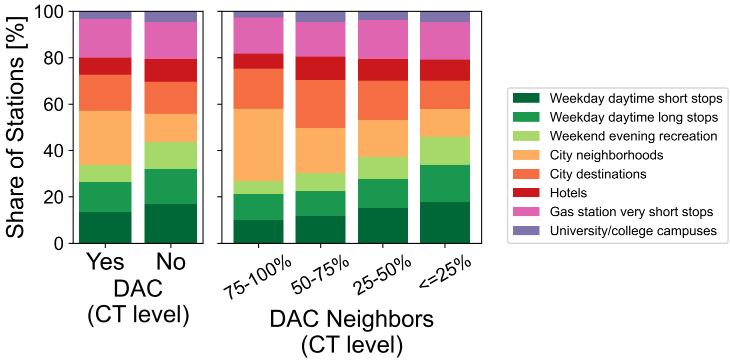

Fig. S20: Share of station clusters by DAC label and by number of DAC neighbors.

Supplementary Note 7: Information on Datasets

Table S9 lists the source of each variable used from the census or ACS data. Table S10 lists the POI categories provided by SafeGraph and shows how they were mapped to the top-level categories used in our analysis. Table S11 shows the number of POIs in the data in each top-level category.

Table S9: Socio-economic data by [15] included

Data Description	Census Bureau Table Number	Table ID
Total population in BG	B01003	B01003e1
Total population in BG: Asian	B02001	B02001e5
Total population in BG: Black	B02001	B02001e3
Total population in BG: Native	B02001	B02001e4
Total population in BG: White	B02001	B02001e2
Total population in BG: Mixed race	B02001	B02001e10
Median household income	B19013	B19013e1
Total number of housing units	B25001	B25001e1
Total number of housing units: Owner-occupied	B25003	B25003e2
Total number of housing units: Renter-occupied	B25003	B25003e3
Number of detached housing units	B25024	B25024e2
Number of attached housing units	B25024	B25024e3
Median number of rooms	B25018	B25018e1
Aggregate number of vehicles by housing unit	B25046	B25046e1
Number of people working out of home	B08134	B08134m1
Total commuting modes	B08301	B08301e1
Total commuting modes: Car	B08301	B08301e2
Total commuting modes: Car only	B08301	B08301e3
Total commuting modes: Public transportation	B08301	B08301e10
Total commuting modes: Bike	B08301	B08301e18
Total commuting modes: Walking	B08301	B08301e19
Total commuting modes: Motorcycle	B08301	B08301e17
Total commuting modes: Taxi	B08301	B08301e16
Total commuting modes: None/working from home	B08301	B08301e21
Total commuting modes: Other	B08301	B08301e20
Aggregate travel time to work	B08135	B08135e1
Commuting time: 5 minutes or less	B08303	B08303e2
Commuting time: 5 to 9 minutes	B08303	B08303e3
Commuting time: 10 to 14 minutes	B08303	B08303e4
Commuting time: 15 to 19 minutes	B08303	B08303e5
Commuting time: 20 to 24 minutes	B08303	B08303e6
Commuting time: 25 to 29 minutes	B08303	B08303e7
Commuting time: 30 to 34 minutes	B08303	B08303e8
Commuting time: 35 to 39 minutes	B08303	B08303e9
Number of people 25 years or older: No schooling	B15003	B15003e2
Commuting time: 40 to 44 minutes	B08303	B08303e10
Commuting time: 45 to 49 minutes	B08303	B08303e11
Number of people 25 years or older: Highschool diploma	B15003	B15003e17
Number of people 25 years or older: Some college education, without degree	B15003	B15003e20
Commuting time: 60 to 89 minutes	B08303	B08303e12
Commuting time: 90 minutes or more	B08303	B08303e13
Number of people 25 years or older: Bachelor's degree	B15003	B15003e22
Number of people 25 years or older: Master's degree	B15003	B15003e23
Number of people 25 years or older: Professional school degree	B15003	B15003e24
Number of people 25 years or older: Doctorate	B15003	B15003e25
Number of people 25 years or older: Associate's degree	B15003	B15003e21
Number of people with Bachelor's degree	B15012	B15012e1
Number of people with Bachelor's degree: Business	B15012	B15012e10
Number of people with Bachelor's degree: Computers Mathematics and Statistics	B15012	B15012e2
Number of people with Bachelor's degree: Biological Agricultural and Environmental Sciences	B15012	B15012e3
Number of people with Bachelor's degree: Physical and Related Sciences	B15012	B15012e4
Number of people with Bachelor's degree: Engineering	B15012	B15012e7
Number of people with Bachelor's degree: Science and Engineering Related Fields	B15012	B15012e9

Table S10: Safegraph POI categories and summarizing categories used in the analysis

Categories Used in Analysis	Top Categories According to Safegraph
Commercial	Automobile Dealers, Automotive Equipment Rental and Leasing, Automotive Parts, Accessories, and Tire Stores, Automotive Repair and Maintenance, Beer, Wine, and Liquor Stores, Book Stores and News Dealers, Building Material and Supplies Dealers, Chemical and Allied Products Merchant Wholesalers, Clothing Stores, Consumer Goods Rental, Death Care Services, Department Stores, Direct Selling Establishments, Drycleaning and Laundry Services, Electronics and Appliance Stores, Employment Services, Florists, Furniture Stores, General Merchandise Stores, including Warehouse Clubs and Supercenters, General Rental Centers, Greenhouse, Nursery, and Floriculture Production, Grocery Stores, Hardware, and Plumbing and Heating Equipment and Supplies Merchant Wholesalers, Health and Personal Care Stores, Home Furnishings Stores, Household Appliances and Electrical and Electronic Goods Merchant Wholesalers, Jewelry, Luggage, and Leather Goods Stores, Lawn and Garden Equipment and Supplies Stores, Lumber and Other Construction Materials Merchant Wholesalers, Machinery, Equipment, and Supplies Merchant Wholesalers, Motor Vehicle and Motor Vehicle Parts and Supplies Merchant Wholesalers, Other Miscellaneous Store Retailers, Other Motor Vehicle Dealers, Other Personal Services, Personal Care Services, Personal and Household Goods Repair and Maintenance, Postal Service, Printing and Related Support Activities, Professional and Commercial Equipment and Supplies Merchant Wholesalers, Services to Buildings and Dwellings, Shoe Stores, Special Food Services, Specialized Design Services, Specialty Food Stores, Sporting Goods, Hobby, and Musical Instrument Stores, Travel Arrangement and Reservation Services, Used Merchandise Stores, Wired and Wireless Telecommunications Carriers, Metal and Mineral (except Petroleum) Merchant Wholesalers, Petroleum and Petroleum Products Merchant Wholesalers, Apparel, Piece Goods, and Notions Merchant Wholesalers, Miscellaneous Durable Goods Merchant Wholesalers, Other Telecommunications, Grocery and Related Product Merchant Wholesalers, Other Support Services, Computer Systems Design and Related Services, Miscellaneous Nondurable Goods Merchant Wholesalers, Farm Product Raw Material Merchant Wholesalers, Furniture and Home Furnishing Merchant Wholesalers, Commercial and Industrial Machinery and Equipment Rental and Leasing, Electronic and Precision Equipment Repair and Maintenance, Couriers and Express Delivery Services, Office Supplies, Stationery, and Gift Stores

Manufacturing	Drugs and Druggists' Sundries Merchant Wholesalers, Foundation, Structure, and Building Exterior Contractors, Warehousing and Storage, Vegetable and Melon Farming, Support Activities for Mining, Electronic Shopping and Mail-Order Houses, Apparel Accessories and Other Apparel Manufacturing, Bakeries and Tortilla Manufacturing, Beverage Manufacturing, Coating, Engraving, Heat Treating, and Allied Activities, Electric Power Generation, Transmission and Distribution, Electrical Equipment Manufacturing, Footwear Manufacturing, Glass and Glass Product Manufacturing, Household Appliance Manufacturing, Motor Vehicle Manufacturing, Natural Gas Distribution, Other Miscellaneous Manufacturing, Other Transportation Equipment Manufacturing, Paint, Coating, and Adhesive Manufacturing, Remediation and Other Waste Management Services, Utility System Construction, Waste Collection, Waste Management and Remediation Services, Waste Treatment and Disposal, Petroleum and Coal Products Manufacturing, Other Food Manufacturing, Ventilation, Heating, Air-Conditioning, and Commercial Refrigeration Equipment Manufacturing, Semiconductor and Other Electronic Component Manufacturing, Architectural and Structural Metals Manufacturing, Other Leather and Allied Product Manufacturing, Aerospace Product and Parts Manufacturing, Clay Product and Refractory Manufacturing, Hardware Manufacturing, Alumina and Aluminum Production and Processing, Agriculture, Construction, and Mining Machinery Manufacturing, Steel Product Manufacturing from Purchased Steel, Navigational, Measuring, Electromedical, and Control Instruments Manufacturing, Converted Paper Product Manufacturing, Plastics Product Manufacturing, Other Animal Production, Medical Equipment and Supplies Manufacturing, Other Fabricated Metal Product Manufacturing, Other Wood Product Manufacturing, Cut and Sew Apparel Manufacturing; Oil and Gas Extraction, Freight Transportation Arrangement, General Freight Trucking, Other Specialty Trade Contractors, Other Support Activities for Transportation, Specialized Freight Trucking, Support Activities for Crop Production, Data Processing, Hosting, and Related Services, Residential Building Construction, Oil and Gas Extraction, Commercial and Industrial Machinery and Equipment (except Automotive and Electronic) Repair and Maintenance, Sugar and Confectionery Product Manufacturing, Highway, Street, and Bridge Construction, Household and Institutional Furniture and Kitchen Cabinet Manufacturing, Machine Shops; Turned Product; and Screw, Nut, and Bolt Manufacturing, Other Nonmetallic Mineral Product Manufacturing

Recreation	Individual and Family Services, Spectator Sports, Motion Picture and Video Industries, Social Assistance, Amusement Parks and Arcades, Gambling Industries, Museums, Historical Sites, and Similar Institutions, Other Amusement and Recreation Industries, Performing Arts Companies, RV (Recreational Vehicle) Parks and Recreational Camps, Religious Organizations, Scenic and Sightseeing Transportation, Scenic and Sightseeing Transportation, Land
Education/Child Care	Child Day Care Services, Colleges, Universities, and Professional Schools, Educational Support Services, Elementary and Secondary Schools, Junior Colleges, Other Schools and Instruction, Technical and Trade Schools, Business Schools and Computer and Management Training
Transit	School and Employee Bus Transportation, Water Transportation, Interurban and Rural Bus Transportation, Support Activities for Air Transportation, Support Activities for Road Transportation, Support Activities for Water Transportation, Taxi and Limousine Service, Transit and Ground Passenger Transportation, Deep Sea, Coastal, and Great Lakes Water Transportation, Other Transit and Ground Passenger Transportation, Rail Transportation, Scheduled Air Transportation, Urban Transit Systems
Gas Station	Gasoline Stations
Medical	General Medical and Surgical Hospitals, Nursing Care Facilities (Skilled Nursing Facilities), Nursing and Residential Care Facilities, Other Ambulatory Health Care Services, Outpatient Care Centers, Psychiatric and Substance Abuse Hospitals, Specialty (except Psychiatric and Substance Abuse) Hospitals, Home Health Care Services, Medical and Diagnostic Laboratories, Offices of Dentists, Offices of Other Health Practitioners, Offices of Physicians, Community Food and Housing, and Emergency and Other Relief Services, Continuing Care Retirement Communities and Assisted Living Facilities for the Elderly, Other Residential Care Facilities, Residential Intellectual and Developmental Disability, Mental Health, and Substance Abuse Facilities

Office	Accounting, Tax Preparation, Bookkeeping, and Payroll Services, Activities Related to Credit Intermediation, Activities Related to Real Estate, Administration of Economic Programs, Administration of Human Resource Programs, Advertising, Public Relations, and Related Services, Agencies, Brokerages, and Other Insurance Related Activities, Architectural, Engineering, and Related Services, Building Equipment Contractors, Building Finishing Contractors, Civic and Social Organizations, Depository Credit Intermediation, Grantmaking and Giving Services, Insurance Carriers, Investigation and Security Services, Justice, Public Order, and Safety Activities, Legal Services, Lessors of Real Estate, Management of Companies and Enterprises, Management, Scientific, and Technical Consulting Services, National Security and International Affairs, Nondepository Credit Intermediation, Offices of Real Estate Agents and Brokers, Other Financial Investment Activities, Other Information Services, Other Investment Pools and Funds, Other Professional, Scientific, and Technical Services, Promoters of Performing Arts, Sports, and Similar Events, Radio and Television Broadcasting, Securities and Commodity Contracts Intermediation and Brokerage, Social Advocacy Organizations, Sound Recording Industries, Business Support Services, Agents and Managers for Artists, Athletes, Entertainers, and Other Public Figures, Administration of Housing Programs, Urban Planning, and Community Development, Executive, Legislative, and Other General Government Support, Scientific Research and Development Services, Business, Professional, Labor, Political, and Similar Organizations, Insurance and Employee Benefit Funds, Independent Artists, Writers, and Performers, Newspaper, Periodical, Book, and Directory Publishers, Cable and Other Subscription Programming, Software Publishers
Hotels	Traveler Accommodation, Rooming and Boarding Houses, Dormitories, and Workers' Camps
Restaurants and Bars	Drinking Places (Alcoholic Beverages), Restaurants and Other Eating Places

Table S11: Number of POIs per category.

	# Points of Interest
Commercial	3889796
Education/Child Care	345317
Gas Stations	159894
Hotels	110351
Manufacturing	439242
Medical	1564966
Office	2711095
Recreation	928866
Restaurants and Bars	1202756
Transit	722171

Supplementary Note 8: Clustering Methods

Choice of K

To select the number of clusters with K-Means clustering, we prepare an elbow plot of the within-cluster sum of squares (inertia) as shown in Figure S21. The inertia will decrease as the number of clusters increases, but there is a trade-off to finding the optimal number of clusters: if we choose too few clusters, the clustering provides little additional information for our analysis; if we choose too many, results are too complex and also add little insight to our analysis. An elbow would indicate a point where the marginal benefit of increasing the number of clusters is smaller. We calculate a numerical approximation of the second derivative to help identify potential elbows, and consider a range of candidate K values with relatively higher “elbow strength”: 4, 5, 7, 8, and 13.

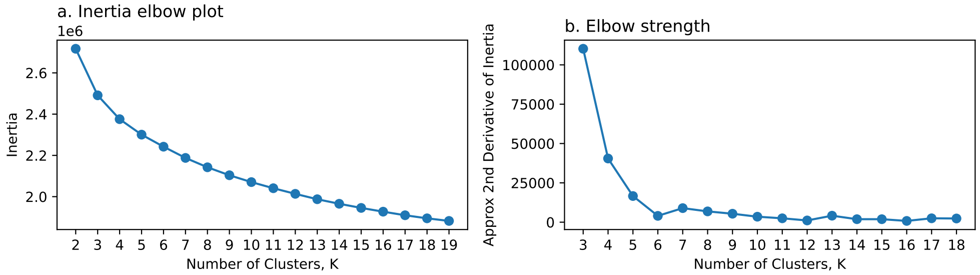

Fig. S21: (a) Elbow plot of clustering inertia. (b) Numerical approximation of the second derivative of the inertia: higher values indicate stronger elbows.

The clusters that appear between the different choices of K are quite consistent, with more refined information with increasing number of clusters. Figure S22 shows how station location clusters in the K=4 case map to station location clusters in the K=8 case. We see that the main types of stations are already visible with K=4: city locations, weekday daytime locations, short stops, and evening weekend locations. However, K=4 hides much of the nuance and many of the insights drawn from K=8, like the distinction between gas stations and other local short stops.

These cluster label interpretations were drawn from plots of the normalized cluster centers values, shown in Figures S23, S24, S25, S26, and S27. Figures S28, S29, S30, and S31 compare the splits between each increasing option for K. We find that K=13 gives too much detail: with too many clusters it is difficult to keep track and draw useful insights in the next steps of the analysis. We instead choose K=8 as the optimal number of clusters, as it has more distinct, interpretable clusters than K=7.

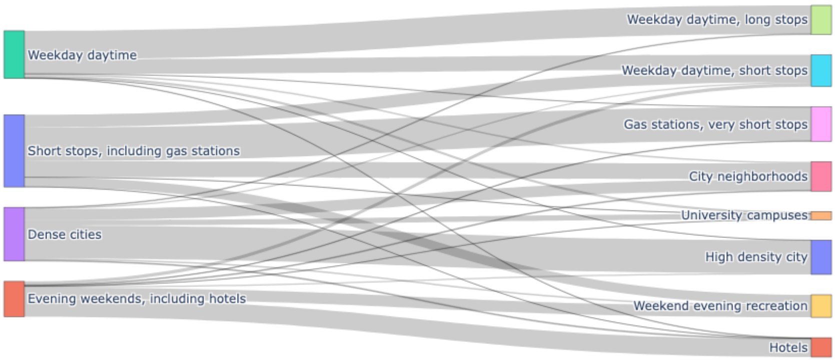

Fig. S22: Comparing the split of stations between K=4 and K=8 clustering.

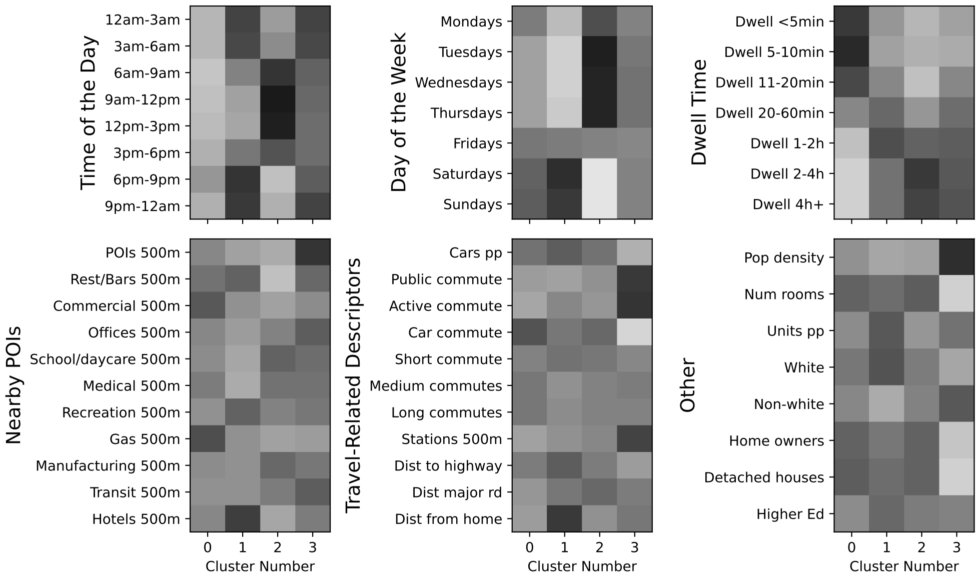

Fig. S23: Illustration of cluster center values for K-Means clustering with K=4.

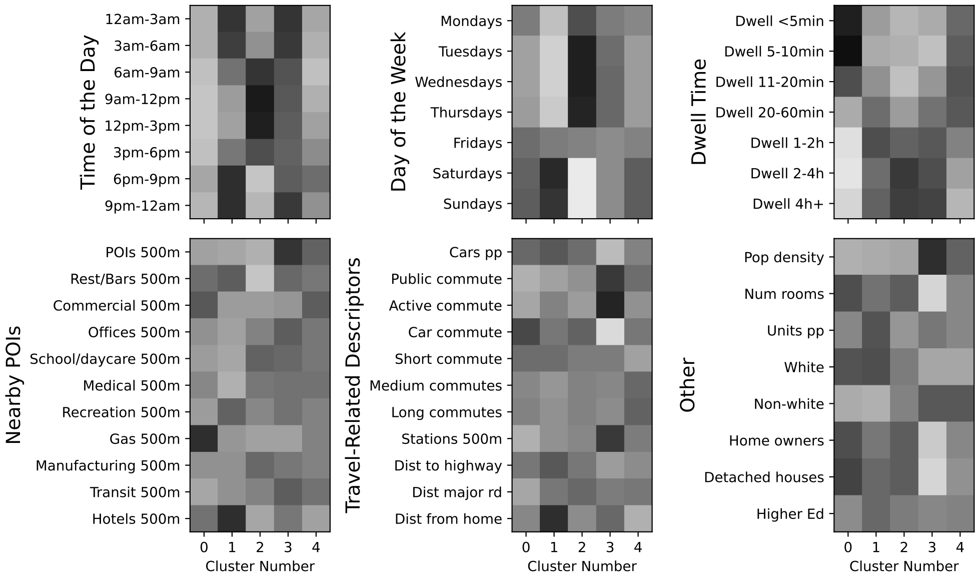

Fig. S24: Illustration of cluster center values for K-Means clustering with K=5.

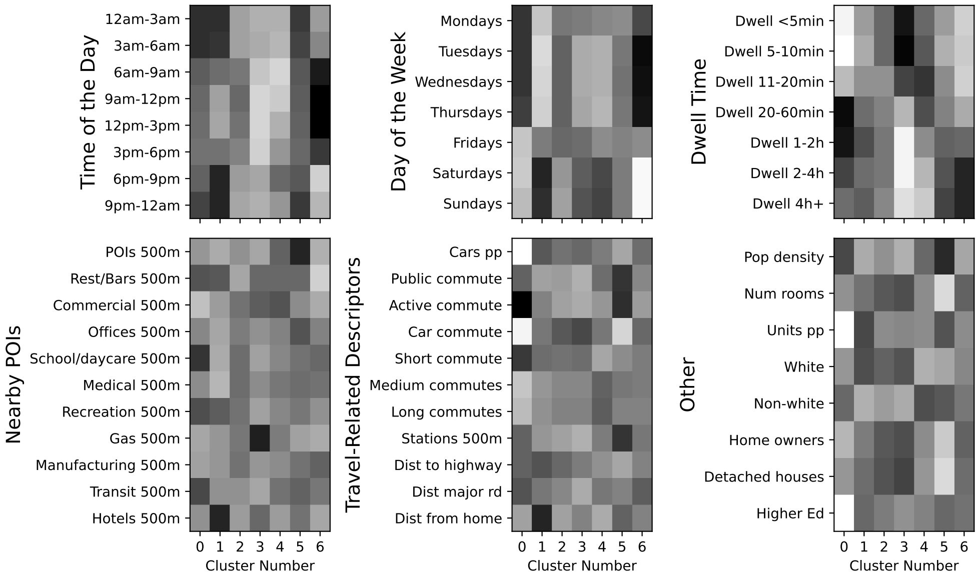

Fig. S25: Illustration of cluster center values for K-Means clustering with K=7.

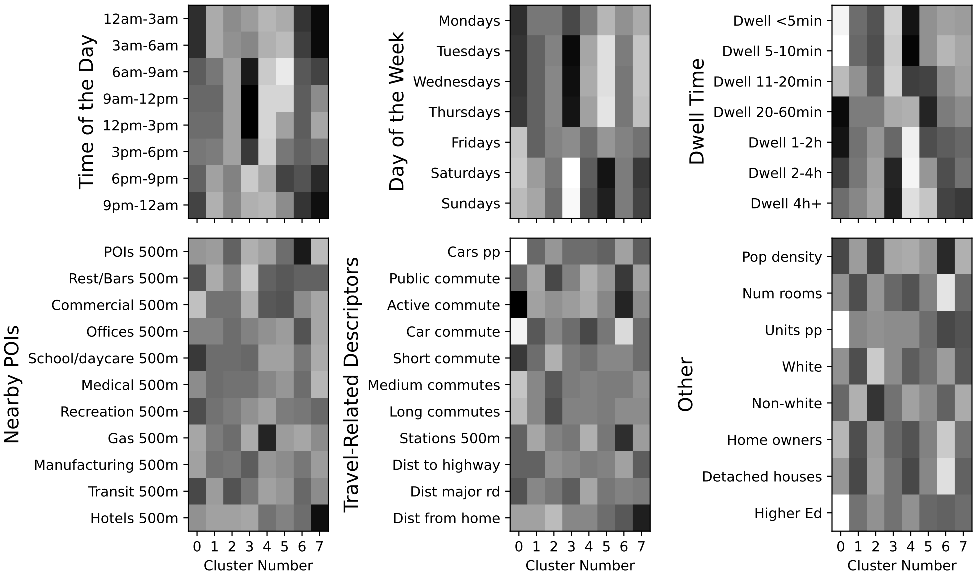

Fig. S26: Illustration of cluster center values for K-Means clustering with K=8.

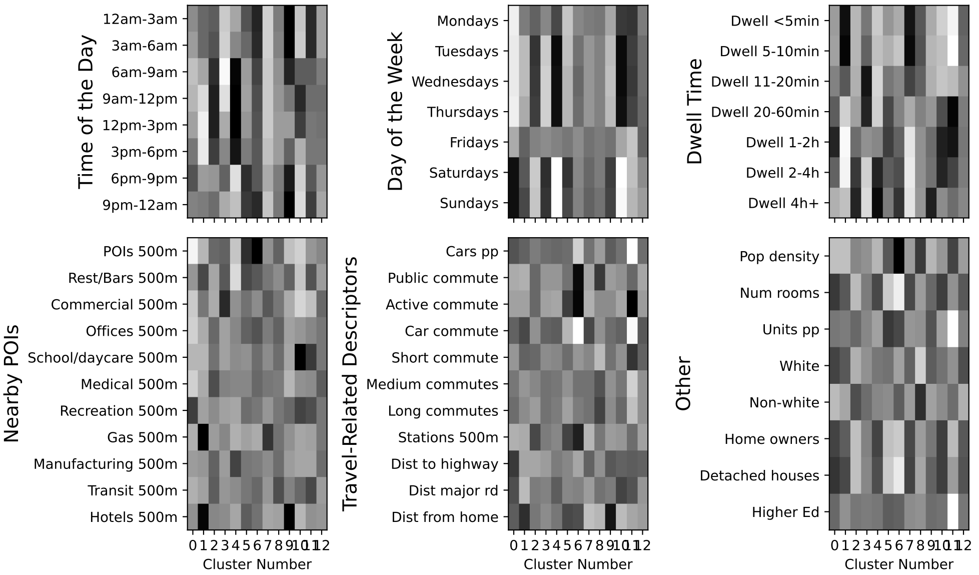

Fig. S27: Illustration of cluster center values for K-Means clustering with K=13.

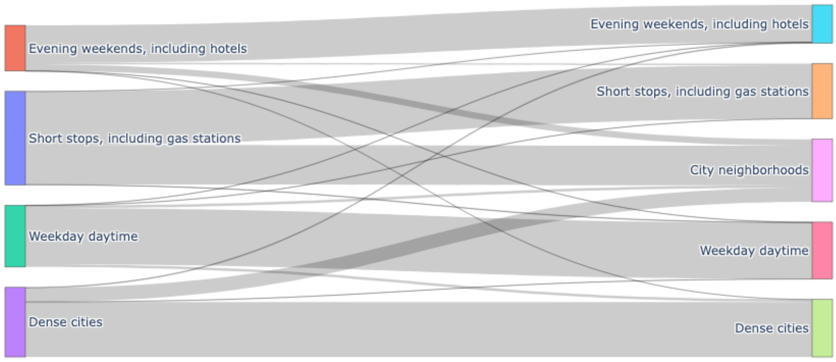

Fig. S28: Comparing the split of stations between K=4 and K=5 clustering.

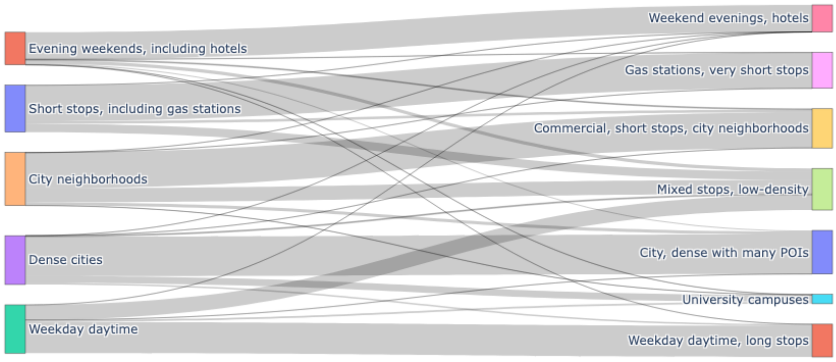

Fig. S29: Comparing the split of stations between K=5 and K=7 clustering.

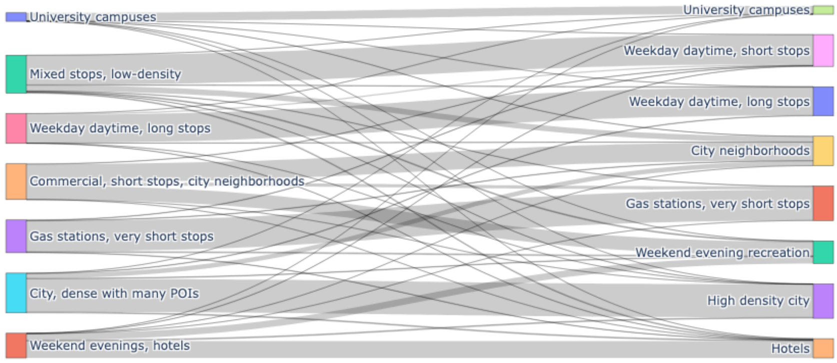

Fig. S30: Comparing the split of stations between K=7 and K=8 clustering.

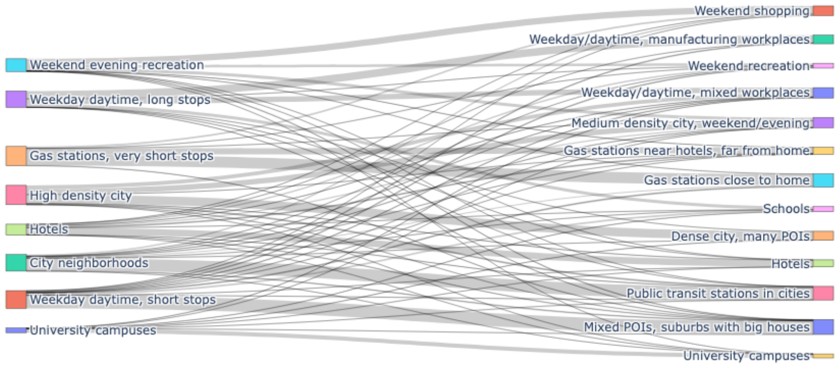

Fig. S31: Comparing the split of stations between K=8 and K=13 clustering.

Comparison with Other Clustering Methods

K-Means benefits from widespread familiarity and thus easier interpretability for readers and policymakers, but the use of K-Means does make implicit assumptions about the structure of the data. To test the robustness of our K-Means-based clustering, we also considered several other clustering methods.

One example alternative is agglomerative clustering with Ward's method [16]. Due to the very large number of stations and input variables, to make the computation feasible we gave the algorithm a pre-computed connectivity matrix based on a graph of each station's 30 nearest neighbors. The resulting clusters can be interpreted based on the cluster centers illustrated in Figure S32 as:

1. Gas stations, very short stops,
2. City destinations,
3. Weekday daytime, long stops,
4. City neighborhoods,
5. Hotels,
6. Weekend evening recreation,
7. Rural recreation sites,
8. University/college campuses.

Compared to our main clustering based on K-Means, the only additional cluster found here is cluster 7, "Rural Recreation Sites". The "Weekday daytime, short stops" main cluster is not a distinct cluster with this method. These differences are relatively minor and we do not expect them to have changed our results in the main paper.

Another example alternative is Gaussian Mixture Modeling (GMM). We note that this is an unusual application of GMM due to the high number of features. The clusters found using GMM with 8 components in the mixture can be interpreted based on the cluster centers illustrated in Figure S33 as:

1. Weekday daytime, long stops,
2. Weekend recreation,
3. Weekday anytime, long stops, including university/college campuses,
4. City destinations,
5. Hotels,
6. Gas stations, very short stops,
7. Weekday daytime, both long and short stops,
8. City neighborhoods and short stops.

Compared to our main clustering based on K-Means, this method shows some differences. Namely, the weekday clusters are separated differently here, not purely based on dwell time and time of day. Our main analysis, however, focuses on the "Gas stations, very short stops" and "Hotels" clusters, which are present with this method as well. We therefore do not expect these differences to have changed our conclusions in the main paper.

With so many initial input variables, we also considered a two step method: first clustering the input columns as a means of dimensionality reduction, then clustering the stations using the composite inputs. Two of the column clustering methods we

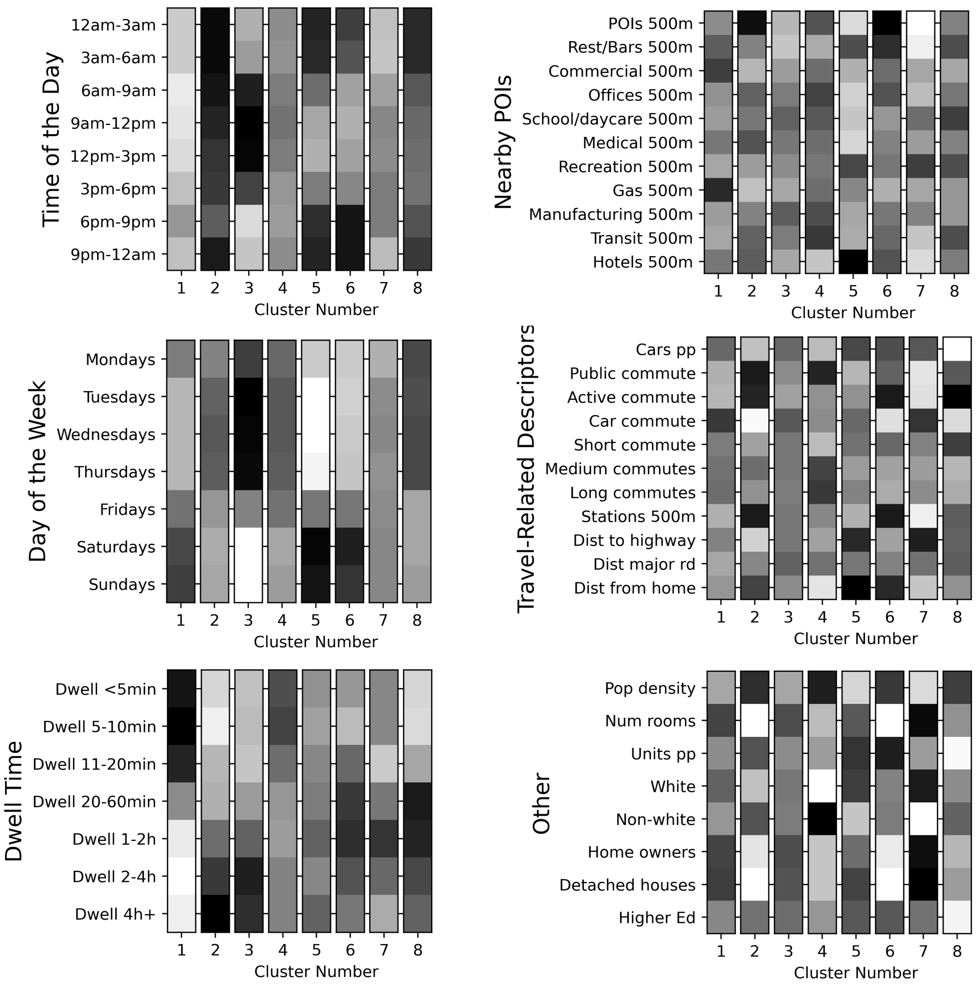

Fig. S32: Heatmap of cluster centers for alternative station clustering using agglomerative clustering with Ward's method. Low values are shown with white and high values are shown with black.

considered are illustrated in Figure S34, variations of agglomerative clustering. In this example, we chose agglomerative clustering with Ward's linkage (Figure S34b) and 8 clusters. We named the composite column clusters "Weekday/daytime", "Dense city", "Weekend/evening, restaurants and shopping", "Car suburbs", "Hotels", "Big houses", "Gas stations", and "Schools and parks". We then projected the data onto these composite clusters and applied K-Means clustering to the station locations. The elbow plot is shown in Figure S35 and the cluster centers for interpretation of K=5 and K=7 are shown in Figure S36. These results largely confirm our findings from the main analysis as many of the familiar clusters appear, but with the composite columns

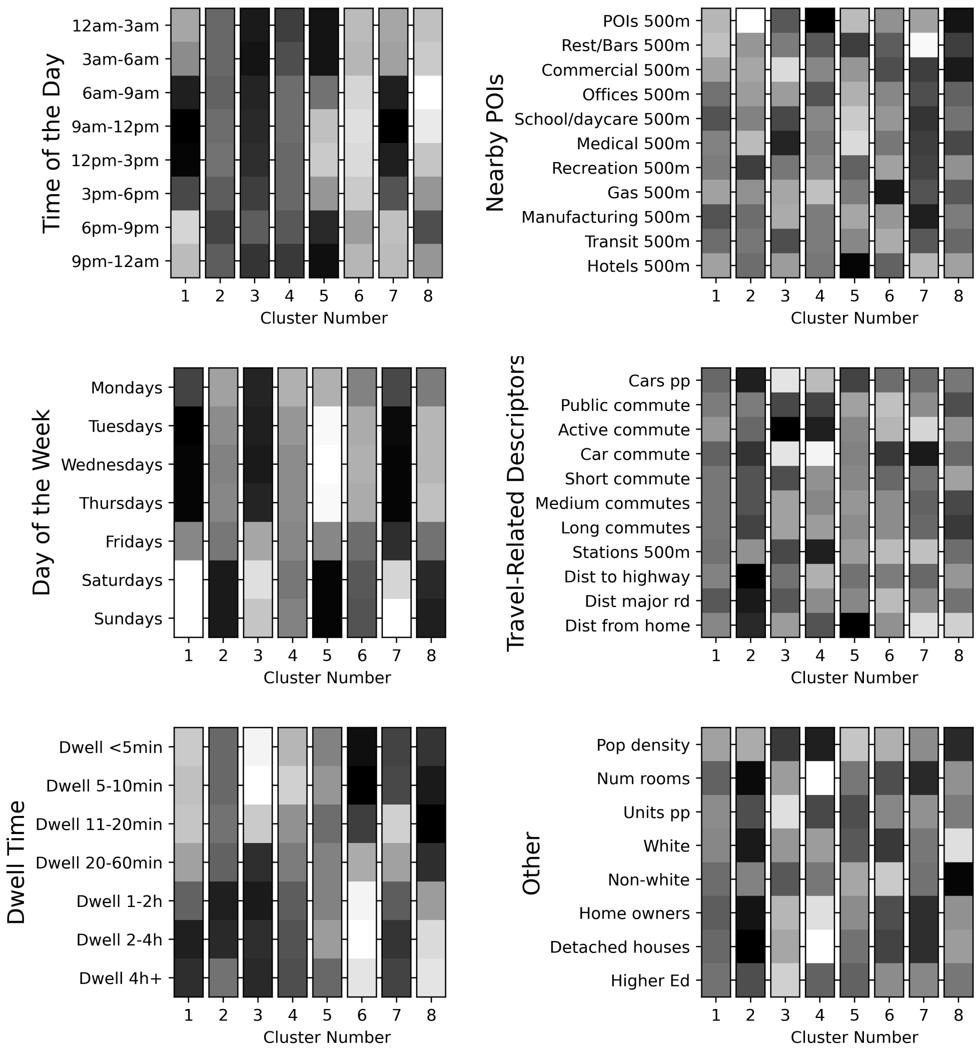

Fig. S33: Heatmap of cluster centers for alternative station clustering using Gaussian Mixture Modeling. Low values are shown with white and high values are shown with black.

they are harder to interpret; we therefore chose to use the simpler one step method for the main paper. We explored several other clustering options but found either that they confirmed our findings or that they were intractable given the large size of the data set.

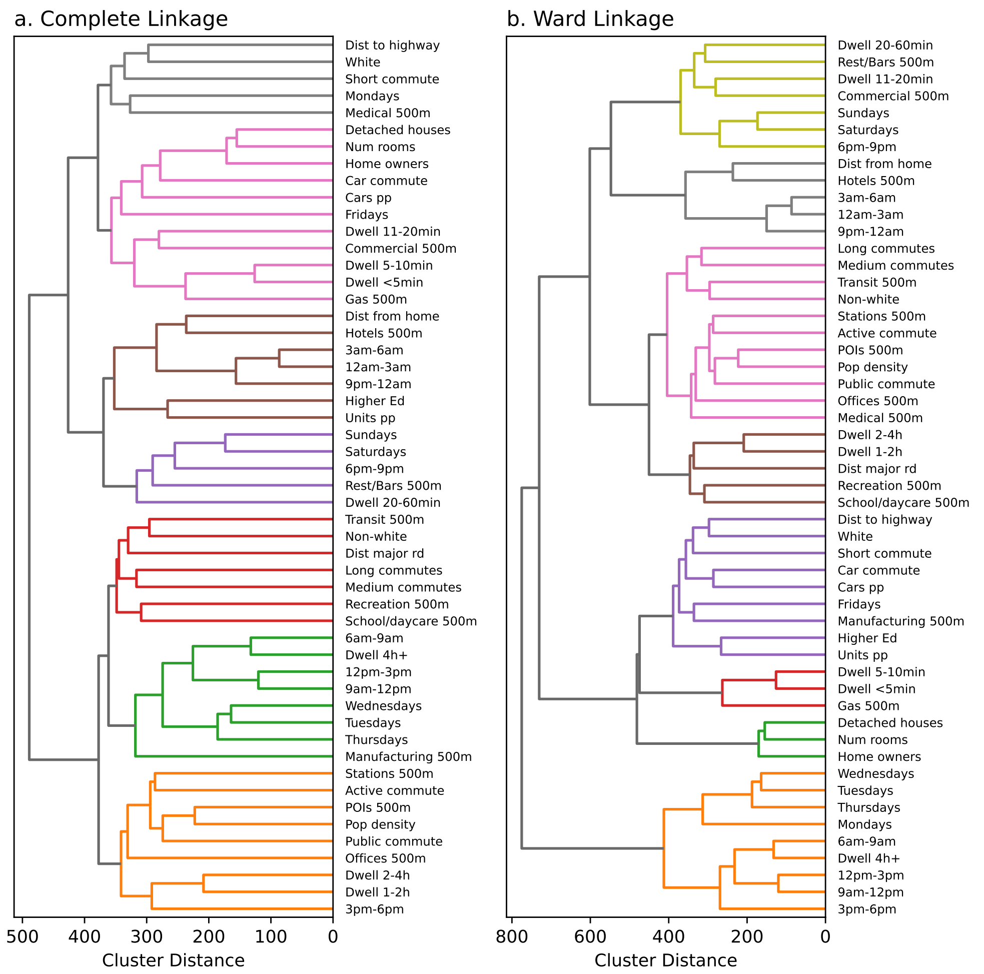

Fig. S34: Alternative clustering method: dendrogram plots of agglomerative column clustering.

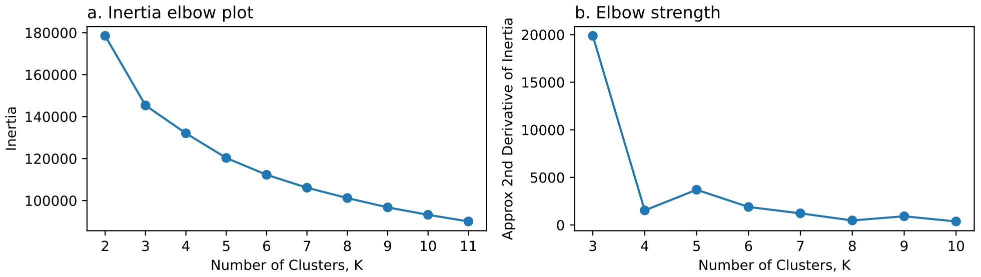

Fig. S35: Alternative clustering method: elbow plots of cluster inertia applying K-Means to composite columns.

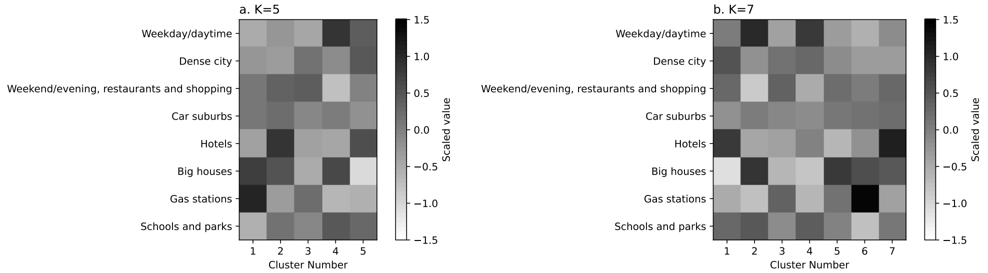

Fig. S36: Alternative clustering method: cluster centers illustrate the interpretation of station location clusters from applying K-Means to the composite columns, with (a) K=5 or (b) K=7 clusters.

References

[1] The White House: Build a Clean Energy Economy: A Guidebook to the Inflation Reduction Act's Investments in Clean Energy and Climate Action. https://www.whitehouse.gov/cleanenergy/inflation-reduction-act-guidebook/ (accessed 2023-07-08) (2023)

[2] The White House: A Guidebook to the Bipartisan Infrastructure Law. https://www.whitehouse.gov/build/guidebook/ (accessed 2024-01-29) (2023)

[3] Hanig, L., Ledna, C., Nock, D., Harper, C.D., Yip, A., Wood, E., Spurlock, C.A.: Finding gaps in the national electric vehicle charging station coverage of the United States. Nature Communications **16**(1), 561 (2025)

[4] White House Council on Environmental Quality: Climate and Economic Justice Screening Tool: Methodology. https://screeningtool.geoplatform.gov/ (accessed 2024-07-17). Data archived at https://climateprogramportal.org/resource/climate-and-economic-justice-screening-tool-cejst/ (2022)

[5] CALeVIP by the California Energy Commission: Fast Charge California Project. https://calevip.org/fast-charge-california-project (accessed 2025-08-08) (2025)

[6] California Energy Commission: California Energy Commission Launches $38 Million Project for EV Charging in Low-Income and Disadvantaged Communities. https://www.energy.ca.gov/news/2023-09/california-energy-commission-launches-38-million-project-ev-charging-low-income (accessed 2025-08-08) (2023)

[7] California Volkswagen Mitigation Trust: Light-Duty Zero-Emission Infrastructure, Electric. https://www.californiavwtrust.org/ev-infrastructure/ (accessed 2026-05-01) (2021)

[8] Sievertson, M.: The LADWP To Expand Free Electric Vehicle Charging Stations In Underserved Communities. https://laist.com/news/climate-environment/the-ladwp-to-expand-free-electric-vehicle-charging-stations-in-underserved-communities (accessed 2025-08-08) (2023)

[9] LADWP: Residential EV Charger Rebate Program. https://www.ladwp.com/residential-services/programs-and-rebates-residential/electric-vehicles/residential-ev-charger-rebate-program (accessed 2025-08-08) (2024)

[10] Qmerit: A Comprehensive Guide to Colorado EV Tax Credits, Rebates & Charging Incentives. https://qmerit.com/location/colorado/ (accessed 2025-08-08) (2025)

[11] Alternative Fuels Data Center: New York Laws and Incentives. https://afdc.energy.gov/laws/all?state=NY (accessed 2025-08-08) (2025)

[12] National Historical Geographic Information System: B19013. https://data2.nhgis.org/main (accessed 2026-05-04) (2014)

[13] National Historical Geographic Information System: Geographic Crosswalks: 2010s Block Groups → 2020s Block Groups. https://www.nhgis.org/geographic-crosswalks#to-block-groups. (accessed 2026-05-04) (2026)

[14] Alternative Fuels Data Center: Federal and State Laws and Incentives. https://afdc.energy.gov/data_download/laws_and_incentives_format (accessed 2025-07-21) (2025)

[15] Safegraph: Census Data & Neighborhood Demographics 2020. https://www.safegraph.com/free-data/open-census-data (accessed 2022-11-22) (2022)

[16] Pedregosa, F., Varoquaux, G., Gramfort, A., Michel, V., Thirion, B., Grisel, O., Blondel, M., Prettenhofer, P., Weiss, R., Dubourg, V., Vanderplas, J., Passos, A., Cournapeau, D., Brucher, M., Perrot, M., Duchesnay, E.: Scikit-learn: Machine learning in Python. Journal of Machine Learning Research **12**, 2825–2830 (2011)
